# Advances in Photodynamic Treatment of Precancerous and Cancerous Gynecological Diseases

**DOI:** 10.3390/cancers17152421

**Published:** 2025-07-22

**Authors:** Polina Alekseeva, Vladimir Makarov, Kanamat Efendiev, Aida Gilyadova, Victor Loschenov

**Affiliations:** 1 Light-Induced Surface Phenomena Department, Natural Sciences Center, Prokhorov General Physics Institute of the Russian Academy of Sciences, 119991 Moscow, Russia; vi.makarov@physics.msu.ru (V.M.); kanamatius@mail.ru (K.E.); loschenov@mail.ru (V.L.); 2 Department of Laser Micro-, Nano-, and Biotechnology, Institute of Engineering Physics for Biomedicine, National Research Nuclear University “MEPhI”, 115409 Moscow, Russia; 3 Department of Oncology, Radiotherapy and Reconstructive Surgery, Levshin Institute of Cluster Oncology, Sechenov First Moscow State Medical University, 119435 Moscow, Russia; aida-benyagueva@mail.ru; 4 Gynecological Department, National Medical Research Treatment and Rehabilitation Center, 125367 Moscow, Russia

**Keywords:** photodynamic therapy, photosensitizers, cervical dysplasia, cervical cancer, human papillomavirus, vulva lichen sclerosus, Langerhans cells, macrophages, immune cells

## Abstract

**Simple Summary:**

Gynecologic cancers, such as cervical and vulvar neoplasms, remain a major global health challenge due to late diagnosis, high recurrence rates, and limited treatment effectiveness. Despite the encouraging potential of photodynamic therapy, several significant challenges must be addressed to fully realize its therapeutic capabilities. The key limitations include its restricted penetration depth, heterogeneous distribution of photosensitizers, and diminished efficacy within hypoxic tumor environments. Furthermore, a critical aspect that often receives insufficient attention is the immune system’s response, which is particularly important in combating human papillomavirus-associated malignancies. This review focuses on how photodynamic therapy can improve gynecologic disease treatments by boosting immune activity and discusses the importance of optimizing the treatment parameters for maximized safety and effectiveness.

**Abstract:**

High mortality rates and poor quality of life result from the late-stage detection and frequent recurrence of gynecological neoplasms. **Background/Objectives**: The aim of this study was to conduct a systematic analysis of the energy parameters of photodynamic therapy (PDT) in the treatment of cervical and vulvar lesions, with a focus on stimulating immune responses leading to human papillomavirus (HPV) eradication and lesion regression without adverse effects, such as thermal damage. **Methods**: A total of 46 peer-reviewed studies published between January 2010 and April 2024 were analyzed. These studies focused on PDT applications for cervical and vulvar lesions, sourced from Google Scholar, Scopus, and Web of Science. **Results**: Although PDT shows promise, significant limitations exist, such as insufficient consideration of individual tumor characteristics, restricted treatment depths, and the heterogeneous distribution and low selectivity of photosensitizer (PS) accumulation in tumors. Tumor hypoxia further reduces PDT’s effectiveness, and most studies overlook immune system activation, which is crucial for targeting HPV infections and improving antitumor responses. **Conclusions**: Advancing the research into PDT’s molecular and cellular mechanisms, optimizing the immune response stimulation, and improving the PS and delivery methods could enhance the safety and effectiveness of cervical and vulvar neoplasm treatments. The use of personalized PDT parameters may reduce the side effects and enhance the outcomes for patients suffering from gynecological diseases.

## 1. Introduction

**Figure 1:**
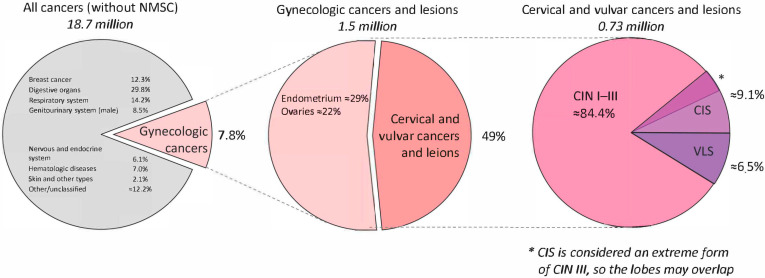
Multistep prevalence breakdown of gynecologic neoplasms relevant to PDT. Abbreviations: PDT—photodynamic therapy.

The high mortality rates and poor quality of life associated with gynecologic malignancies represent serious global public health challenges [1]. These issues stem from the late-stage detection of the disease, its rapid progression, and its high recurrence rates, all of which significantly reduce the chances of successful treatment and patient survival [2]. By 2030, the World Health Organization (WHO) aims to achieve 90% human papillomavirus (HPV) vaccination coverage, conduct screening in 70% of the target population, and ensure adequate treatment for 90% of cases as part of its global strategy to accelerate the elimination of cervical cancer. To reach these aims, novel therapeutic approaches are required to reduce the recurrence risk and improve patient outcomes. According to WHO data, the incidence and mortality rates of gynecologic malignancies are continuing to rise globally. In 2022, more than 660,000 women were diagnosed with cervical cancer and nearly 350,000 women died from cervical cancer (Figure 1) [2].

The first pie chart (left) shows the global distribution of all cancer cases excluding non-melanoma skin cancer. The middle chart highlights the proportions of gynecologic cancers, including cervical, endometrial, ovarian, vulvar, and vaginal malignancies. The final chart (right) zooms in on the cervical and vulvar precancerous conditions specifically discussed in this review—CIN I–III, CIS, and VLS. CIS is often classified as an advanced stage of CIN III; thus, it overlaps with CIN in its clinical categorization.

HPV, particularly high-risk (HR) types such as HPV 16 and HPV 18, is the primary risk factor for cervical cancer [3,4,5]. Overall, the global prevalence of HPV in cervical carcinomas is estimated at 99.7% [6].

The immune system effectively clears various HPV types in most infected women, yet in approximately 15% of cases, the infection persists, significantly increasing the risk of cancer development [4,7,8,9]. This persistence is a result of HPV’s ability to evade immune surveillance and establish chronic infections. A key mechanism of immune evasion involves the suppression of Langerhans cells (LCs) [4,5,7,8,10,11,12,13,14]. Under normal conditions, LCs play a crucial role in maintaining immune surveillance by detecting pathogens, including HPV, at epithelial entry points, while also preventing autoimmune responses through immunoregulatory mechanisms. LCs not only recognize pathogens but also activate T lymphocytes, initiating an adaptive immune response. However, HR-HPV types (16, 18, 31, 33, 35, 39, 45, 51, 52, 56, 58, 59, and 66) have been shown to disrupt LC function [15].

This disruption creates an immunosuppressive tumor microenvironment in the cervical epithelium, which is particularly evident in high-grade lesions and cervical cancer. The microenvironment is characterized by various cellular phenotypes producing the anti-inflammatory cytokine interleukin-10 (IL-10), which facilitates persistent HPV infection and tumor progression. Previous studies have demonstrated increased production of IL-10 by keratinocytes, macrophages, and LCs in high-grade cervical lesions and cancer, with this effect being more pronounced in patients over 30 years old and those with a high viral load of HR-HPV [13]. This finding supports the hypothesis that HPV actively employs immunosuppressive strategies to maintain infection, contributing to the absence of an effective immune response in some individuals [4,5,7,8,10,11,12,13,14].

Local immune dysfunction [16,17,18], characterized by an altered LC density in the epidermis [17,19,20] and dysregulated T-lymphocyte populations in the dermis [19,21,22,23], plays a significant role in the pathogenesis of vulvar lichen sclerosus (VLS) [20]. Studies have shown that early-stage lesions contain a higher number of LCs than late-stage lesions [20,24,25]. Additionally, the amount of inflammatory infiltrates decreases in advanced VLS, suggesting LC suppression contributes to immune escape. IL-10 secretion by epithelial cells has been implicated in reducing antigen presentation by LCs and transforming them into inactive phenotypes [26,27]. Chronic inflammation in VLS leads to oxidative damage to lipids, deoxyribonucleic acid (DNA), and proteins [28], further increasing the risk of vulvar cancer development [16,25,29,30].

The treatment of gynecologic precancerous and cancerous diseases, including cervical, endometrial, vaginal, and vulvar lesions, depends on the disease stage and typically involves surgery, radiation therapy, and chemotherapy, either as standalone treatments or in combination. The staging is determined clinically based on the tumor size, local spread, and imaging assessments [31,32,33,34,35,36,37,38,39,40,41,42,43].

All conventional therapeutic modalities carry a significant risk of complications, including damage to surrounding tissues, impaired lymphatic drainage leading to lymphedema, and a considerable reduction in patient quality of life. Furthermore, the high recurrence rate, particularly in advanced-stage malignancies, remains a major challenge. These limitations underscore the need for innovative treatment strategies that enhance the therapeutic efficacy while minimizing the adverse effects. Among such promising strategies are photodynamic therapy (PDT) and immunotherapy [44,45,46,47,48,49,50].

PDT is an emerging, minimally invasive, organ-preserving treatment for gynecologic malignancies. It is based on the interaction of three key components:

1. A light-sensitive compound known as a photosensitizer (PS);
2. Light of a wavelength that matches the absorption peak of the PS;
3. Molecular oxygen.

Before PDT, the PS accumulates in tumor tissues, especially in blood vessels and immune cells, due to the enhanced permeability and retention (EPR) effect.

The most commonly used photosensitizers (PSs) in PDT for gynecologic cancers are 5-aminolevulinic acid (5-ALA) and its derivatives, as well as chlorin e6 (Ce6). The mechanisms of photodynamic tumor destruction differ significantly between 5-ALA and Ce6, impacting the overall PDT efficacy [51]. Furthermore, 5-ALA induces the accumulation of protoporphyrin IX (PpIX) in tumor cells due to a deficiency of ferrochelatase, an enzyme responsible for converting PpIX into heme [52]. In contrast, Ce6 primarily accumulates in the tumor vasculature, where it readily diffuses into surrounding tumor tissues due to increased vascular permeability [53,54]. After sufficient PS accumulation, the tumor is exposed to light irradiation [55].

When light is absorbed, the photosensitizer creates reactive oxygen species (ROS) such as singlet oxygen that damage cancer cells [56]. ROS-induced oxidative stress damages cellular structures, including lipids and proteins. Due to deficiencies in antioxidant defense mechanisms, tumor cells are particularly susceptible to ROS-induced damage, resulting in apoptosis, necrosis, or autophagy-mediated cell death [57,58,59,60]. Additionally, PDT damages tumor-associated blood vessels, depriving the tumor of oxygen supply [61], and modulates immune responses by either polarizing tumor-associated macrophages (TAMs) from an immunosuppressive M2 phenotype to a pro-inflammatory M1 phenotype [62] or deactivating TAMs, which play a crucial role in immune evasion [62,63].

PDT activates T-cell-mediated immune responses. It engages Langerhans cells, dendritic cells, and plasmacytoid dendritic cells. This enhances the antigen presentation and boosts antitumor immunity [64,65,66]. Furthermore, LC activation promotes cytokine production [67], which enhances inflammation and recruits additional immune cells that contribute to antigen uptake and presentation, amplifying the immune response [67,68,69,70,71,72,73]. By modulating the immune and cellular pathways involved in tumor progression, PDT presents a promising therapeutic strategy for precancerous and cancerous gynecologic diseases [44].

The aim of the study was to provide a comprehensive synthesis of the existing knowledge, focusing on the PDT energy parameters (energy density and power density of laser irradiation) that effectively stimulate immune responses leading to HPV eradication and precancerous and cancerous lesion regression without causing thermal or necrotic damage to superficial lesion layers.

## 2. Materials and Methods

A comprehensive search was conducted on the Google Scholar, Scopus, and Web of Science databases to identify relevant research studies. The systematic review included 46 articles published between January 2010 and August 2024 that employed PDT for precancerous or cancerous diseases of the cervix or vulva (Table S1). The main terms of the search strategy were: “photodynamic therapy”, “cervical cancer”, “cervical dysplasia”, “human papillomavirus, “vulva lichen sclerosus”, “Langerhans cells”, “immune cells”, “macrophages”.

The complete search strategy was: (“photodynamic therapy” [tw] OR “PDT” [tw] OR “photodynamic exposure” [tw] OR “photodynamic” [tw]) AND (“photosensitizer” [tw] OR “PS” [tw] OR “5-aminolevulinic acid” [tw] OR “5-ALA” [tw] OR “Hexaminolevulinate” [tw] OR “HAL” [tw] OR “Methylaminolaevulinate” [tw] OR “MAL” [tw] OR “Chlorin e6” [tw] OR “Ce6” [tw] OR “Photofrin” [tw] OR “Photogem” [tw]) AND (“cervical cancer” [tw] OR “CIS” [tw] OR “cervical neoplasms” [tw] OR “cervical dysplasia” [tw] OR “Cervical Intraepithelial Neoplasia” [tw] OR “CIN” [tw] OR “cervical tumor” [tw] OR “cervical lesion” [tw] OR “cervical disease” [tw]) OR (“vulvar neoplasms” [tw] OR “vulva lichen sclerosus” [tw] OR “VLS” OR “vulvar disease” [tw] OR “vulvar lesion” [tw]) AND (“Langerhans cells” [tw] OR “immune cells” [tw] OR “macrophages” [tw] OR “TAM” [tw] OR “T lymphocytes” [tw] OR “immune response” [tw] OR “tumor microenvironment” [tw]) AND (“treatment outcome” [tw] OR “complete response” [tw] OR “CR” [tw] OR “partial response” [tw] OR “PR” [tw] OR “neoplasm recurrence” [tw] OR “disease recurrence” [tw] OR “lesion recurrence” [tw] OR “residual lesion” [tw] OR “persistent infection” [tw] OR “human papillomavirus clearance” [tw] OR “HPV clearance” [tw] OR “immune activation” [tw] OR “inflammatory response” [tw] OR “treatment effectiveness” [tw] OR “treatment safety” [tw]).

**Figure 2:**
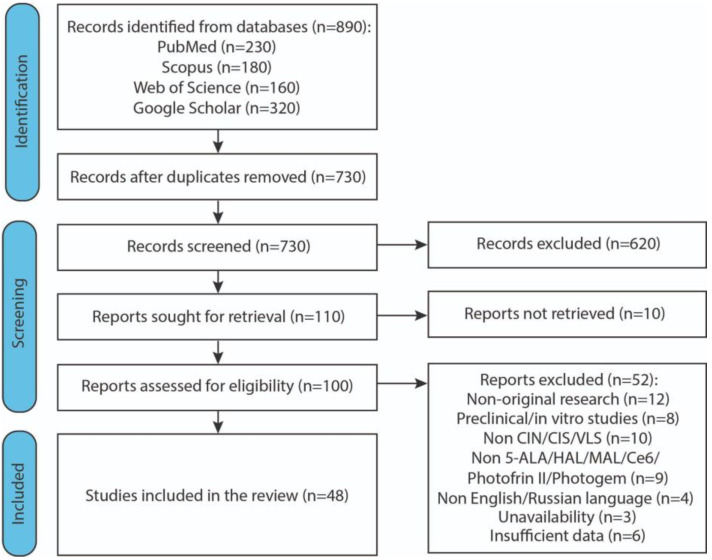
Flow diagram illustrating the study selection process. Abbreviations: CIN—cervical intraepithelial neoplasia; CIS—carcinoma in situ; VLS—vulvar lichen sclerosus; 5-ALA—5-aminolevulinic acid; HAL—hexaminolevulinate; MAL—methylaminolaevulinate; Ce6—chlorin e6.

Initially, 890 articles were identified. After the screening stage, 46 studies were included in the final systematic analysis. Figure 2 reports the flow diagram of the study selection process.

To ensure the relevance and scientific rigor of this systematic review, the following inclusion and exclusion criteria were applied.

Inclusion criteria:

1. Study design: Original research articles, including prospective or retrospective clinical studies, case series with any number of patients, and conference abstracts containing the necessary methodological and clinical data.
2. Population: Studies involving female patients diagnosed with cervical intraepithelial neoplasia (CIN), carcinoma in situ (CIS), or VLS.
3. Intervention: Application of photodynamic therapy (PDT) using the PSs 5-ALA, hexaminolevulinate (HAL), methylaminolaevulinate (MAL), Ce6, Photofrin II, or Photogem.
4. Outcome measures: Reported data on at least one of the following: HPV clearance, lesion regression, complete response (CR) or partial response (PR), or immune response indicators.
5. Language and availability: Published in English or Russian and available in full-text format.

Exclusion criteria:

1. Non-original research: Reviews, insufficiently informative conference abstracts, editorials, letters to the editor, dissertations, and book chapters.
2. Non-human studies: Preclinical or in vitro studies not involving human subjects.
3. Insufficient data: Studies lacking essential outcome data or a detailed methodology for replication or quality assessment.
4. Language: Studies not published in English or Russian.

For each included study, the following data were extracted: year, country, lesion localizations, HPV types, numbers of patients, PS concentration, accumulation time, light wavelength, power and energy densities, exposure time, repetition rate, number of courses, diagnostic methods, and HPV and lesion outcomes. All data were summarized descriptively in structured tables.

## 3. Results and Discussion

### 3.1. Cervical Tumors

#### 3.1.1. 5-ALA

PDT in conjunction with 5-ALA has been proven to be a safe and effective non-invasive treatment for HPV-associated gynecological cancers. The method is associated with minimal side effects, the preservation of normal tissue structures, and no effect on the reproductive function of women. The 5-ALA-PDT modality finds application in both inpatient and outpatient settings [74,75], and is considered a valuable addition to the armamentarium of contemporary oncological treatments.

A comprehensive review of the extant literature yielded 11 articles that investigated the efficacy and safety of 5-ALA PDT for the treatment of CIN grades I, II, and III; carcinoma in situ (CIS); and cervical condylomata acuminata (CCA) associated with low-risk (LR-) HPV and HR-HPV, including types 6, 11, 16, 18, 31, 59, and others (Table 1).

Tissues of the cervical canal, cervix, and vagina that had accumulated complete 5-ALA-induced PpIX were irradiated with red light sources exhibiting wavelengths of 630, 632.8, and 635 nm or a broadband source of 590–760 nm for 20 to 50 min, with the duration being contingent upon the dimensions of the lesions. For cervical irradiation, an optical fiber with an end radiator was used to deliver light uniformly over the entire surface of the cervix, and for cervical and vaginal irradiation, an optical fiber with a diffuser was used to deliver light uniformly over the entire length of the treated area. According to the literature, the radiant power density rates varied from 100 to 204 mW/cm^2^ and the energy density rates from 100 to 120 J/cm^2^. The patients participated in between two and ten courses of PDT, with intervals of one to two weeks between each course. The evaluation of PDT’s efficacy was predominantly conducted through the utilization of diagnostic methodologies, encompassing a clinical evaluation, biopsy, histology, cytology, colposcopy, electron microscopy, and polymerase chain reaction (PCR) for HPV. The patients were subsequently followed up for a period of 3–24 months.

A summary of the results showed that in previous studies [77,78,79,80,81], 5-ALA PDT achieved a complete cure in more than 90% of patients with CCA, CIN, and cervical cancer caused by HPV after 3–4 PDT sessions. In contrast, the remaining studies [75,76,82,83,84] reported CR rates of less than 90% among patients. The article [85] did not provide quantitative data on the treatment response. However, one study [80] reported HPV eradication rates greater than 90% following four courses of PDT, while other studies [75,76,78,79,81,82,83,84,85] reported rates of less than 90%. Another study [77] did not provide HPV elimination results.

The high efficacy and safety of 5-ALA-PDT, as well as its pronounced antiviral effect, were confirmed by the results of a previous study [76], which included 5 patients diagnosed with CIN II or CIN III caused by HPV. All patients with CIN II demonstrated a CR to treatment within a period of 9 months, while 1 patient with CIN III remained HPV-positive for a duration of 6 months following the completion of 3 or 4 treatment courses. Another study [79] involved 76 patients with CIN I and persistent cervical HR-HPV infection randomly divided into two groups. The treatment group received 3 courses of 5-ALA–PDT at two-week intervals and the control group did not receive any treatment. At three months, the HR-HPV remission rats were 64.10% in the treatment group and 24.32% in the control group. At nine months, these rates improved to 76.92% and 32.40%, respectively. Among patients with CIN I, 5 in the treatment group showed complete recovery at nine months, while no such cases were observed in the control group. The efficacy and safety of 5-ALA-PDT were evaluated in the treatment of CIN and persistent HR-HPV in 54 patients [82]. The patients were divided into three groups—group 1 with CIN III, group 2 with CIN I, and group 3 with simple HR-HPV infection. After 6 treatment sessions, the remission rates were 71.43% for simple HR-HPV, 63.64% for CIN I, and 50%for CIN III. The histological remission rates were 80% for simple HR-HPV, 69.57% for CIN I, and 75% for CIN III.

The 5-ALA-PDT procedure is characterized by its ease of use, which contributes to a swift recovery post-procedure. This method also has minimal side effects and allows the preservation of normal tissue structures of the cervix. Furthermore, 5-ALA PDT has been shown to have minimal complications, such as cervical stenosis, and does not affect a woman’s reproductive function. The method is considered safe for repeated use, provided strict adherence to all established guidelines is maintained. The aforementioned characteristics of 5-ALA-PDT have led to its demonstration of the possibility of wide application in both hospital and outpatient settings. In a previous study [80], which included 110 patients with CIN I, the clinical efficacy of two treatment methods—local 5-ALA-PDT and high-frequency electro–ionotherapy—was assessed. Six and nine months after the last treatment, the 5-ALA PDT group showed 81.81% and 10.91% remission rates for HR-DNA HPV, respectively. In contrast, the control group showed 52.73% remission at 6 months and 7.27% at 9 months. The PDT group showed higher remission rates and total remission numbers than the control group, with statistically significant differences. The 5-ALA PDT approach also facilitated fertility preservation in women diagnosed with CIN III [84]. A study encompassing 96 patients with histologically confirmed CIN III and highly oncogenic HPV types, conducted three months following 5-ALA PDT, exhibited an overall lesion regression rate of 89.58% and an HPV elimination rate of 79.17%.

PDT has effectively overcome the problems of frequent recurrence and scarring of the cervix, which have been serious obstacles in the treatment of CCA [77]. A study of 48 CCA patients showed that a single course of PDT eradicated cervical lesions in 62.5% of women. After three procedures, the CR rate reached 95.8%. No recurrence was observed in 95.6% of patients after 12 months. Electron microscopy revealed that 5-ALA-PDT targeted proliferative keratinocytes without significant harm to surrounding normal tissues. A retrospective study [83] of 31 patients with CIN II and HR-HPV showed that 77.78% caused complete tumor clearance at 12 months, with partial tumor regression confirmed via histology. Only 7.41% maintained CIN II, and no patients progressed to CIN III or cancer. The overall HPV remission rate was 62.96%.

The present study investigated the safety and efficacy of PDT with alpha-lipoic acid in the elimination of cervical HPV infection and CCA [78]. The study involved 56 women with CCA on the cervix and external genitalia. The genotyping identified HPV subtypes 6, 11, 16, and 18. After 1–4 treatment sessions, 98.2% of cases showed complete lesion remission, with 83.9% achieving HPV clearance. In 10 cases, a single treatment eliminated lesions and eradicated HPV. The relapse rate was 3.6%.

**Figure 3:**
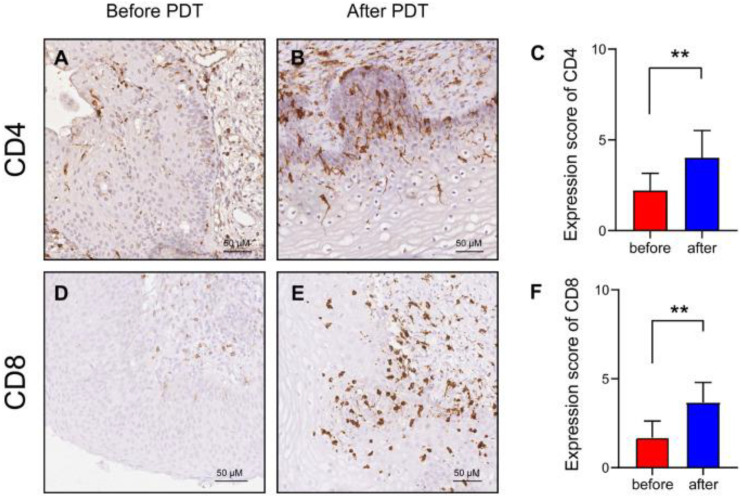
Immunohistochemistry detection before and after PDT in patients with CIN II: (**A**,**B**) CD4+ T cell distribution before and after PDT; (**C**) CD4+ T cell expression in 22 patients; (**D**,**E**) CD8+ T cell distribution before and after PDT; (**F**) CD8+ T cell expression in 22 patients (** *p* ˂ 0.01). Abbreviations: PDT—photodynamic therapy [75].

PDT employing 5-ALA has been shown to elicit two distinct effects—direct destruction of tumor cells through the generation of ROS and substantial impact on the immune response [86]. This can increase the number of antigen-specific T cells that enhance the immunogenic efficacy of PDT [87,88]. The optimal PDT parameters, such as the concentration of the PS, the wavelength of the radiation, and the energy density, significantly impact the LC activation. For instance, when utilizing 5-ALA, which results in PpIX accumulation in tumor cells, photodynamic exposure leads to cell HAL destruction and the release of tumor antigens [89]. This, in turn, stimulates LCs, which interact with antigens through receptors such as langerin (CD207) and activates a cascade of cytokines including IL-12 and TNF-α. A substantial body of research has demonstrated the efficacy of PDT in activating the immune system and promoting T cell proliferation in patients with CIN [90]. A retrospective study [75] involving 22 patients with CIN II, CIN III, and HR-HPV demonstrated that 5-ALA PDT increased the numbers of CD4+ and CD8+ T cells, suggesting that the specific cellular immunity was enhanced by PDT to eliminate cervical lesions. Figure 3 shows that the expression of CD4+ and CD8+ T cells significantly increased after PDT (*p* < 0.01). There was no significant difference between the CD4/CD8 ratios before and after PDT (*p* = 0.063).

At 3 months post-5-ALA PDT, the mean lesion clearance rate was 81.82% and the HPV clearance rate was 54.55%. Six months later, the CIN clearance rate was 90.91% and the HPV clearance rate was 86.36%.

#### 3.1.2. HAL and MAL

The use of 5-ALA derivatives, HAL and MAL, for PDT offers significant advantages compared to traditional therapeutic methods employing 5-ALA, making this approach safer, more effective, and more convenient for patients. HAL and MAL exhibit greater specificity toward affected tissues than 5-ALA. This is due to their faster penetration through cellular membranes and their preferential accumulation in pathological cells, thereby minimizing their impact on healthy tissues. Compared to 5-ALA, both MAL and HAL have demonstrated an improved tissue penetration depth and high treatment efficacy [91]. HAL- and MAL-PDT are associated with fewer side effects compared to 5-ALA. This reduces the risk of developing allergic reactions, painful sensations, and other adverse events [92].

As a result of the literature review, 6 articles were selected that investigated the efficacy and safety of the 5-ALA-PDT method for the treatment of CIN I, II, and III, and CIS associated with HR-HPV, such as types 16 and 18, among others (Table 2).

For HAL- and MAL-PDT, light sources emitting wavelengths of 629, 630, and 633 nm were used. The power density rates of the light irradiation varied from 25 to 120 mW/cm^2^, and the energy density rates ranged from 25 to 150 J/cm^2^. The durations of photodynamic exposure ranged from 17 to 276 min. The patients completed one to two courses of PDT, administered once every 4 weeks. The treatment efficacy was assessed using diagnostic methods such as blood sampling, biopsy and histology, cytology, colposcopy, HPV PCR, and fluorescence imaging. Follow-up examinations were conducted at 3, 6, and 12 months after PDT.

An analysis of the results showed that PDT achieved a CR in more than 90% of patients with CIN I and CIN II in one study [96] after one PDT session with a 20% HAL solution, and in another study [97] after one to two courses of therapy with a 5% MAL solution. In other studies [93,94,95], a CR was achieved in less than 90% of patients. In one study [90], quantitative data on treatment outcomes were not provided. In all reviewed studies, the efficacy rate of HPV elimination was less than 90%. In several articles [90,93,96], there was no information regarding HPV eradication.

PDT using HAL demonstrated high efficacy and safety, offering a promising alternative to observation and surgical procedures in patients with CIN. One study [94] included 67 patients with CIN I–III. Furthermore, 84% of the patients were initially HR-HPV positive. The patients were divided into six treatment groups using HAL- and MAL-PDT. The CR rate was 17% with HAL 10 mM, 33% with HAL 40 mM (3 h application), and 35% with MAL. A 3 h HAL 40 mM application showed a higher CR rate (43%) compared to a 12 h application (13%). In the second part, using HAL 40 mM and a 3 h application at 50 J/cm^2^, the CR rate was 33%, matching the overall 100 J/cm^2^ group result. The CR rate for patients with CIN I and II was higher than for those with CIN III. Overall, 57% of the patients with CIN II/III achieved complete or partial remission at 50–100 J/cm^2^. Among 27 patients with complete remission, 21 had positive HPV tests at the start, and 14 of them achieved complete HPV remission. In a prospective, double-blind, phase IIa study [95] involving 70 patients with CIN I, the subjects were randomized into three groups: group 1—vaginal HAL suppositories; group 2—vaginal placebo suppositories; group 3—control. HR-HPV infection was detected in 43% of patients. At 6 months, complete remission occurred in 57% of HAL-PDT patients and 25% of controls, a significant difference (*p* = 0.04). HR-HPV was eradicated in 73% of HAL-PDT patients compared to 50% in controls, although the result was not significant due to the small sample size and high spontaneous clearance rates. No significant difference in CIN lesion response was observed at 3 months between groups.

HAL- and MAL-PDT did not cause irreversible damage to the normal tissues of the cervix. In one study [93] of 25 patients with CIN I–III, macroscopic cervical changes were not observed, and the histological examination showed no signs of apoptosis, necrosis, irritation, vascular changes, or fibrosis six months after HAL- and MAL-PDT. Six months post-PDT, 64% showed either a complete or partial response. No significant differences were found between HAL- or MAL-PDT or different HAL dosages (10 mM vs. 40 mM). The inflammation scores before and after PDT were 1.64 (95% CI: 1.44–1.84) and 1.84 (95% CI: 1.61–2.07), respectively, with no significant change overall, except for one case where the inflammation increased from mild to severe.

The formation and accumulation of PpIX occurs more efficiently in altered and neoplastic cells than in normal cells [98]. The PpIX molecule is characterized by a high fluorescence capacity. This means that upon energy absorption and transition to an excited state, it is capable of fluorescing. Monitoring the fluorescence intensity of PpIX during PDT using methods such as fluorescence spectroscopy or imaging allows for an evaluation of PpIX accumulation and photobleaching in tumor tissue. In one study [96], 23 patients with CIN I/II, either HPV-infected or not, participated. The patients were divided into two groups: Group 1 with CIN I and Group 2 with CIN II. The patients were exposed to PDT with MAL. For the HAL-PDT control, fluorescent images of PpIX were recorded before and after PDT using a probing laser emitting at 400 nm. The results after PDT showed that in all 23 patients who underwent treatment, complete remission was achieved, meaning that CIN completely disappeared. The Papanicolaou smear also did not detect any presence of CIN I or II, thereby obviating the need for a biopsy.

HAL-PDT demonstrated high efficacy in patients with CIN II, including those with HR-HPV, but not in patients with CIN I [97]. A double-blind, randomized, placebo-controlled dose-finding study included a total of 262 women with CIN I/II. HR-HPV infections were observed in 49% of patients with CIN I and 83% of patients with CIN II. The patients were treated with PDT with HAL and a placebo ointment. As a result of the treatment, no statistically significant differences were observed between the CIN I group and the placebo group, or between the combined CIN I/II group and the placebo group. In the study conducted among women with CIN II, a clear dose-dependent effect was observed 3 months after treatment; in the group treated with HAL 5%, the percentage of positive responses was 95% compared to 57% in the placebo group (*p* < 0.001). Among patients with CIN II, in the HAL 5% group, sustained elimination of HPV types 16 and 18 was achieved in 83% of cases both 3 and 6 months after the final PDT course. Meanwhile, in the placebo group, this indicator was only 0% at 3 months and 33% at 6 months.

PDT using HAL stimulates the patient’s immune system, particularly T cell proliferation, which may contribute to the fight against HPV in patients with CIN. In one study [90], two patients participated, one of whom underwent 2 courses of PDT, while the other received a placebo course. After HAL-PDT, measurable changes in CD4+ and CD8+ cells were observed in CIN. In contrast to the patient who received the placebo, in whom a decreased proliferation of T cells in response to HPV16-L1 antigens was observed, the patient treated with HAL-PDT exhibited a significant increase in T cell proliferative activity, especially 90 days after PDT compared to before PDT. This may indicate continuous accumulation of anti-HPV16-L1-reactive T cells following PDT.

Studies show that the use of HAL and MAL creates a local pro-inflammatory microenvironment that stimulates the migration of LCs and their interaction with other components of the immune system, including macrophages and cytotoxic T lymphocytes [99].

PDT parameters, such as the concentration of HAL or MAL, light wavelength, and energy density, play an important role in modulating this effect. For example, an optimal wavelength (approximately 635 nm) and appropriate energy density promote enhanced LC activation and subsequent recruitment of immune cells to the site of damage [100]. This contributes not only to the local destruction of tumor cells but also to the formation of a systemic antitumor immune response.

These results underscore the promising potential of using HAL and MAL within PDT to enhance both local and systemic immune responses in the treatment of gynecologic malignancies.

#### 3.1.3. Ce6

Within the review, 8 articles were analyzed in which PDT with Ce6 was applied for the treatment of CIN I, II, and III; CIS; and microinvasive cancer (MIC)-associated with LR-HPV and HR-HPV, such as types 6, 11, 16, 18, 31, 33, 35, 45, 52, 56, and others (Table 3).

For PDT using Ce6, laser light sources emitting at wavelengths of 660, 661, 662, or 670 nm were used. In the studies we reviewed [101,103,104,105], the exposure parameters for the cervix and the cervical canal were identical. A power density greater than 200 mW/cm^2^ was reported in only one of the cited studies [101], while in the other studies this parameter was not specified. Energy density rates of 100 and 400 mW/cm^2^ were reported in previous studies [101,105], whereas the authors of the other studies did not provide this information. However, in other studies [102,107,108], the irradiation parameters for the cervix and the cervical canal differed. The power density rates for the cervix were 290 and 300 J/cm^2^, while for the cervical canal they ranged from 200 to 250 mW/cm^2^. The energy density rates for the cervix varied from 100 to 350 J/cm^2^, and for the cervical canal from 100 to 250 J/cm^2^. Only in one study [106] were the power density rates different for the cervix and the cervical canal, being 290 and 250 mW/cm^2^, respectively, while the energy density rates did not differ and ranged from 100 to 250 J/cm^2^. The exposure time was reported in only one article [101], which was 20 min. In all studies, the cervix was irradiated using an optical fiber equipped with a microlens for more uniform light distribution in the irradiation field, and the cervical canal was irradiated along its entire length using an optical fiber with a cylindrical diffuser. To assess the efficacy of PDT, several diagnostic methods were used, including blood sampling, biopsy and histology, cytology, colposcopy, PCR analyses of HPV, and video- and spectral-fluorescence diagnostics. After PDT, follow-up examinations were conducted at 1, 3, 6, and 12 months to evaluate the recurrence rate.

After PDT using Ce6, studies [101,102,105,106] achieved complete remission in more than 90% of patients with CIN I–III after 1–3 courses, while one study [103] required 4–8 courses of therapy. In other studies [104,107,108], a complete treatment response was achieved in less than 90% of patients. The HPV eradication outcomes were significantly better than achieving a CR to treatment. All [102,103,104,105,106,107,108] but one study [101] achieved HPV eradication efficacies greater than 90%.

**Figure 4:**
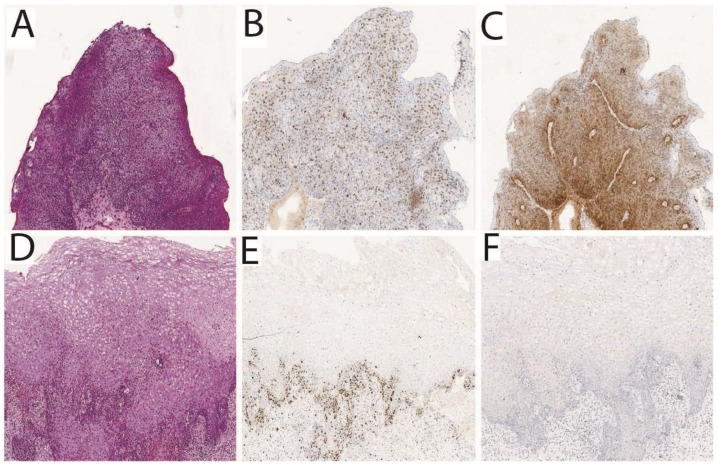
Results of the histological analysis of the cervical tissue samples taken from a patient with CIN III: (**A**–**C**) tumor before PDT; (**D**–**F**) tumor after PDT [106].

PDT using Ce6 demonstrated its efficacy and safety in the treatment of precancerous and cancerous diseases of the cervix. Figure 4 shows the results of the histological analysis of the cervical tissue samples taken from a patient with CIN III before PDT (Figure 4A) and 1 month after PDT with Ce6 (Figure 4D). The absence of the morphological picture of CIN after PDT indicates a CR to therapy. The figure also demonstrates samples of immunohistochemical staining for the Ki67 (Figure 4B,E) and p16 (Figure 4C,F) markers before and after PDT, respectively. After treatment, the color intensity significantly decreases, indicating reduced expression of Ki67 and p16 markers in cervical epithelium [106].

This method selectively targets tumor cells while minimizing the risk of damage to healthy tissues. It allowed for the preservation of the anatomical and functional integrity of the female reproductive organs, which is important for a woman’s fertility [106].

In one study [101], 112 patients with CIN II, CIN III, and CIS were included. In 78.6% of women, HR-HPV was detected before treatment. After treatment, 92.8% showed complete remission confirmed by morphology. At 3 months, 53.4% tested HPV-negative via PCR. However, this rate decreased over time, reaching 29.4% at the 3-year follow-up. The efficacy of PDT was evaluated in 74 patients with early cervical cancer, for whom PDT was offered as an alternative to surgical intervention [103,104]. The patients were divided into two groups based on the type of transformation zone and tumor localization: group 1—ectocervix (type I–II); group 2—endocervix (type III). HR-HPV was detected in 84% of women. After PDT, 84% of group 1 and 88% of group 2 had normal Papanicolaou smears. At 3 months post-PDT, 9.1% tested positive for HPV. No negative changes were observed in the Papanicolaou smears at 6 and 12 months, with HPV detected in 2.8% of group 1 and 3.2% of group 2 patients. No recurrences were recorded over 4.5 years. Three women delivered healthy children.

**Figure 5:**
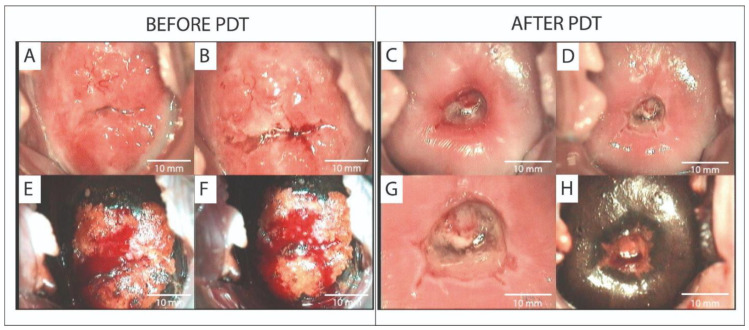
Abnormal and normal colposcopic images in a patient with CIS before PDT (**A**,**B**,**E**,**F**) and 4 weeks after PDT (**C**,**D**,**G**,**H**): (**A**,**C**) native image; (**B**,**D**) cervix treated with 3.5% acetic acid solution; (**E**–**H**) cervix treated with Lugol’s solution—Schiller’s test. Abbreviations: CIS—carcinoma in situ; PDT—photodynamic therapy [107].

Spectroscopic and video-fluorescence methods were employed to monitor Ce6 photobleaching, thereby improving the effectiveness of PDT in treating precancerous and cervical conditions. One study [102] encompassed 10 patients who had been diagnosed with cervical leukoplakia, ranging from CIN I to CIN III, as well as CIS, based on a histological analysis. Before PDT, PCR revealed the presence of HPV strains 16, 18, 6, and 11 in all patients. The administration period of PS was 3 h. Before and after PDT, video- and spectral-fluorescence diagnostics was employed to delineate the boundaries of neoplasms. Three months after PDT, biopsy samples from the cervical region showed complete remission of pathological tissues in all patients, confirming successful treatment. In 9 of 10 patients, the HPV and CIN signs disappeared. In the remaining patient, repeat PDT reduced CIN III to CIN I. After repeated sessions, there was no sign of HPV or CIN in the patient. Another study [106] assessed the efficacy of Ce6-PDT utilizing video- and spectral-fluorescence diagnostics in a cohort of 52 patients with CIN I–III, CIS, microinvasive SCC, and SCC. The presence of oncogenic HPV was confirmed in all patients. After the first PDT course, 80.8% patients showed lesion regression. A PCR analysis of cervical canal secretions revealed that 48 out of 52 patients did not possess the previously identified HPV. PDT enhanced the colposcope’s appearance and reduced the expression of oncogenesis markers in cervical tissue. In another study [107], 45 patients with HPV-related CIN III and CIS were treated with Ce6-PDT. The use of video- and spectral-fluorescence diagnostics made it possible to assess the accumulation and photobleaching of the PS. After PDT, 88.2% of CIN III and 89.3% of CIS patients achieved complete remission, with partial regression in 11.8% and 10.7% of patients, respectively, and significant reductions in Ki-67 and p16 expression. Figure 5 shows the patient’s colposcopic images before and after PDT.

PDT with Ce6 fluorescence control showed higher efficacy and safety in treating HPV-associated CIN III and CIS compared to standard methods [108]. Three months after PDT, 4.4% of the patients had confirmed intraepithelial lesions, while the remaining 95.2% had a normal cytological profile. After 6, 9, and 12 months, all patients remained healthy. In the conization group, 89.8% of patients had CIN II after 3 months, 10.2% had CIN I, and the remaining 77.6% had a normal profile after 12 months. In the PDT group, HPV disappeared in 91.1% of patients and remained in 8.9% with a decrease in viral load. In the conization group, HPV was not detected in 69.4% of patients and it remained in 30.6%. The HPV level in the PDT group was statistically lower (*p* = 0.003). After a second course of PDT, HPV disappeared in all patients, whereas in the conization group, it was detected in 32.7% during the year. The patients who planned their pregnancy had the best reproductive results in the PDT group.

PDT is an effective method for the treatment of precancerous cervical diseases. However, despite its effectiveness, it requires regular follow-up and may lead to recurrences. A retrospective analysis of the efficacy of PDT for early-stage cervical cancer, as well as HPV elimination, was conducted on 28 patients [105]. The cytological examination revealed various stages of precancerous cervical conditions, with a high proportion of CIN III and IV cases. HPV testing showed that 53.6% of patients had confirmed HPV infection. HPV elimination was observed in 82% of cases within a 3 month period after PDT. Among the analyzed cases, complete HPV remission was observed in more than 90% of cases. The probability of the absence of cervical cancer recurrence was 0.8 (95% CI: 0.53–1) at 60 months. The patients experienced mild (35.7%) and severe (28.6%) leukocytic reactions within 3 months after PDT.

The activation of the antitumor immune response during PDT using Ce6 is generally similar to the mechanisms observed in PDT with 5-ALA, HAL, and MAL. The common process involves the destruction of tumor cells, the release of tumor-associated antigens (TAAs), and damage-associated molecular patterns (DAMPs), which activate the innate immune system and initiate an adaptive antitumor response [109].

However, there are key differences between these photosynthesizers that affect the nature of the immune response:

1. Ce6 exhibits high lipophilicity, which ensures better penetration into tumor tissues and increases the selectivity of accumulation in the tumor [110], while 5-ALA, HAL, and MAL are hydrophilic molecules that require metabolic activation to form PpIX. HAL and MAL penetrate the epithelium better due to their lipophilic structure compared to 5-ALA.
2. Ce6 is activated by light over a wavelength range of 660–670 nm, which allows for deeper tissue penetration and the treatment of larger tumors, while 5-ALA, HAL, and MAL are activated by light over approximately 630–635 nm, which limits their penetration depth [111].
3. Ce6 induces a more pronounced destruction of the tumor vascular network, leading to local hypoxia and enhanced inflammation. This promotes additional activation of immune cells. In contrast, 5-ALA, HAL, and MAL are less aggressive in affecting the vascular network, making them more suitable for organ-preserving treatment.
4. Due to its deep penetration and high phototoxicity, Ce6 is more effective in inducing a systemic antitumor immune response, including the activation of memory T cells, resulting in the induction of a systemic effect.

PDT with 5-ALA, HAL, and MAL is more commonly used for localized lesions and has a less pronounced effect on the systemic immune response. Thus, Ce6 stands out among other PSs due to its deeper penetration, high phototoxicity, and ability to induce a systemic antitumor effect. However, 5-ALA, HAL, and MAL remain important tools for the treatment of superficial and localized lesions due to their organ-preserving effects.

#### 3.1.4. Other PS

PDT using the PSs Photofrin II and Photogem represents an alternative method for the treatment of recurrent gynecologic neoplasms, as well as precancerous conditions of the cervix. The application of these PSs ensures the selective elimination of tumor cells while minimizing damage to healthy tissues. PDT with Photofrin II and Photogem demonstrates high efficacy in achieving CR in patients with various gynecologic diseases, while preserving the reproductive function and reducing the risk of recurrences [112].

Within the review, two articles were examined in which PDT with these PSs, namely Photofrin II and Photogem, was applied for the treatment of CIN I, II, and III; CIS; and adenocarcinoma in situ (AIS) associated with LR-HPV and HR-HPV, such as types 16, 18, 33, 58, 66, and others (Table 4).

Photofrin II and Photogem, which are derivatives of hematoporphyrin, were administered systemically to patients at a concentration of 2 mg/kg via intravenous infusion. Approximately 48 h after injection, the cervix and the endocervical canal of the patients were irradiated with laser light at a wavelength of 630 nm. In the study using Photogem, the power density of the light irradiation was 150 mW/cm^2^, the energy density was 150 J/cm^2^, and the exposure time was 3 min. In the study with Photofrin II, the irradiation parameters for the tumor tissue were not provided. The therapeutic efficacy was evaluated using biopsy and histology, cytology, colposcopy, and HPV detection.

The results for the use of PDT in the treatment of 32 patients with recurrent cervical, vulvar, vaginal, ovarian, and endometrial cancers, as well as one case of recurrent anal Paget’s disease, are presented [113]. In 24% patients a complete response was achieved, with a mean response time of 28 months. HPV eradication data were not reported.

PDT combined with or without loop electrosurgical excision procedure (LEEP)–conization may represent a potential alternative for the effective conservative treatment of CIN [114]. A study included 59 patients with CIN II/III who wished to preserve their fertility. The patients were divided into four groups: group 1—PDT only, without LEEP; group 2—PDT combined with LEEP–cone; group 3—PDT administered within 3 months after LEEP–cone due to a positive margin; group 4—PDT administered due to recurrence of CIN at least 12 months after LEEP–cone. After one year of follow-up, 98.1% achieved complete remission, excluding six patients who experienced pregnancy loss. One recurrence occurred after a year, and another case was considered complete remission after three months of follow-up despite the presence of residual disease. At 3 and 12 months post-PDT, 89.8% and 87.0% were HPV DNA-negative, respectively. Among 29 patients who attempted conception, 18 succeeded.

PDT using Photofrin II and Photogem represents a class of first-generation PSs used in PDT for various tumor types. Their primary mechanism of action involves the generation of ROS upon irradiation with light at approximately 630 nm. However, their properties and effects have certain features and limitations that distinguish them from 5-ALA, HAL, MAL, Ce6, and second-generation PSs.

Photofrin II is a mixture of hematoporphyrin oligomers, whereas Photogem is based on purified forms of porphyrin, which improves its photochemical activity. These PSs exhibit lower tumor selectivity and require a longer clearance time, thereby increasing the risk of skin phototoxicity. In addition, their activation wavelength limits the depth of light penetration, making them less effective for treating large or deeply located tumors, in contrast to Ce6, which is activated by light with a wavelength range of 660–670 nm.

The main mechanism of accumulation of Photofrin II and Photogem in the tumor is related to the passive effect of EPR, whereas Ce6 and second-generation PSs often have modifications that increase their selectivity. This leads to more targeted accumulation in tumor tissues and less impact on healthy cells [115].

In terms of immunomodulation, Photofrin II and Photogem are also inferior. Despite their ability to induce the release of DAMPs, such as HSP70 and HMGB1, their impact on adaptive immunity and the activation of cytotoxic T cells is less pronounced. In contrast, Ce6 and second-generation PSs demonstrate more significant destruction of the tumor microenvironment, enhancing both local and systemic immune responses [116].

Moreover, the prolonged clearance time of Photofrin II and Photogem limits their use in patients requiring multiple treatment sessions and increases the risk of skin phototoxicity. Modern PSs, such as Ce6, do not have this disadvantage, as they are cleared more rapidly from the body and exhibit lower toxicity.

Thus, Photofrin II and Photogem have historical significance and continue to be used in clinical practice but modern second-generation PSs and Ce6 provide higher efficacy, selectivity, and safety rates.

A comprehensive analysis of the treatment outcomes for CCA, various degrees of dysplasia, and cervical cancer using PDT with 5-ALA showed complete remission in more than 90% of patients in 5 of 11 articles after 3–4 PDT sessions, with HAL and MAL achieving this in 2 of 6 cases after 1–2 therapy courses, Ce6 in 5 of 8 cases after 1–8 courses, and Photofrin II and Photogem in 1 of 2 cases after 1 course of PDT. The analysis also indicated that 5-ALA, HAL, and MAL are not effective in terms of HPV eradication in CCA, various degrees of dysplasia, and cervical cancer. Only in 1 of 11 studies with 5-ALA was HPV eradication achieved in more than 90% of patients. In none of the 6 studies reviewed with HAL or MAL and Photofrin II or Photogem did the HPV elimination rate reach 90%. The results of PDT using Ce6 were significantly better with respect to HPV than those using 5-ALA, HAL or MAL, and Photofrin II or Photogem. In 7 of 8 studies, HPV elimination was achieved in more than 90% of patients.

The heterogeneity of the treatment results for CCA, various degrees of dysplasia, and cervical cancer using PDT (with less than 90% CR) in patients with these conditions using different PSs such as 5-ALA, HAL, MAL, and Ce6 may be attributed to the following factors:

1. Differences in PDT protocols:
  a PS concentration (5–20% for 5-ALA; 10 mM, 40 mM, 100 mg, 0.2–5% for HAL; 1.2 M, 160 mg/g, 20% for MAL; 0.8–2.5 mg/kg for Ce6; 2 mg/kg for Photofrin II/Photogem);
  b Exposure time (2–5 h for 5-ALA; 3–12 h for HAL/MAL; 2–4 h for Ce6, 48 h for Photofrin II/Photogem);
  c Light irradiation parameters (power density range of 25 to 300 mW/cm^2^, energy density range of 25 to 400 J/cm^2^);
  d Number of treatment sessions (from 1 to 10).
2. Patient characteristics:
  a Disease stage (CIN I, II, III, CIS, MIC);
  b HPV type (LR- and HR-strains);
  c Immune response;
  d Comorbidities (autoimmune diseases or oncological processes).
3. Methodological factors:
  a Diagnostic methods and criteria for evaluating efficacy (biopsy, histology, cytology, colposcopy, HPV PCR, etc.);
  b Duration of patient follow-up (from 3 months to several years);
  c Dosimetry methods (presence or absence of fluorescence diagnostics).

### 3.2. Vulvar Lichen Sclerosus

#### 3.2.1. 5-ALA

5-ALA-PDT is a well-tolerated and effective treatment modality for VLS, leading to the apoptotic death of lymphocytes and keratinocytes, as well as alterations in the levels of cytokines and matrix metalloproteinases that participate in skin remodeling processes. Although the precise mechanism of action of PDT in the treatment of VLS remains incompletely understood, it is postulated that the primary therapeutic effect is directed toward the elimination of sclerotic changes in the skin [117].

A systematic review of the literature included 20 articles that examined the safety and efficacy of 5-ALA-based PDT in the treatment of VLS (Table 5).

After the application period, PDT of the affected zones was performed using light sources emitting at wavelengths of 540, 590–760, 630, 633, 635, or 750, 580–1400 nm. The power density rates of the light irradiation varied among studies from 40 to 204 mW/cm^2^, while the energy density rates ranged from 60 to 150 J/cm^2^ and the exposure times varied from 2 to 40 min. In patients with VLS, PDT was generally administered once every 1–2 weeks. In one study, however, the second treatment session was performed after 4 weeks, the third after 6 months from the initiation of therapy, the fourth after 6 weeks, and subsequently once every 3–4 months [119]. Overall, the patients required between 2 and 10 PDT sessions.

To evaluate the efficacy of PDT, various methods were employed, including questionnaires, assessments of typical clinical symptoms and signs, clinical diagnoses and evaluations, biopsy and histology, colposcopy, vulvoscopy, dermoscopy, peripheral blood sampling, HPV detection, the horizontal Visual Analogue Scale (VAS), the Female Sexual Function Index (FSFI), the Dermatology Life Quality Index (DLQI), high-frequency ultrasound (HFUS), and photodynamic diagnostics (PDD). Following the PDT sessions, follow-up examinations were conducted at 1, 3, 6, and 12 months to assess the recurrence rate.

Treatment with 5-ALA-PDT resulted in significant improvements in patient conditions, a reduction in the intensity of symptoms, and in some cases complete remission of VLS. In several studies, a CR rate exceeding 90% was achieved [81,119,123,124,125,126,127,128,134], whereas in other investigations, the efficacy did not reach the 90% threshold [85,117,118,120,121,122,129,130,131,132,133] Data on the results of HPV eradication were provided in only two studies, and the eradication rates did not exceed 90% [81,129].

**Figure 6:**
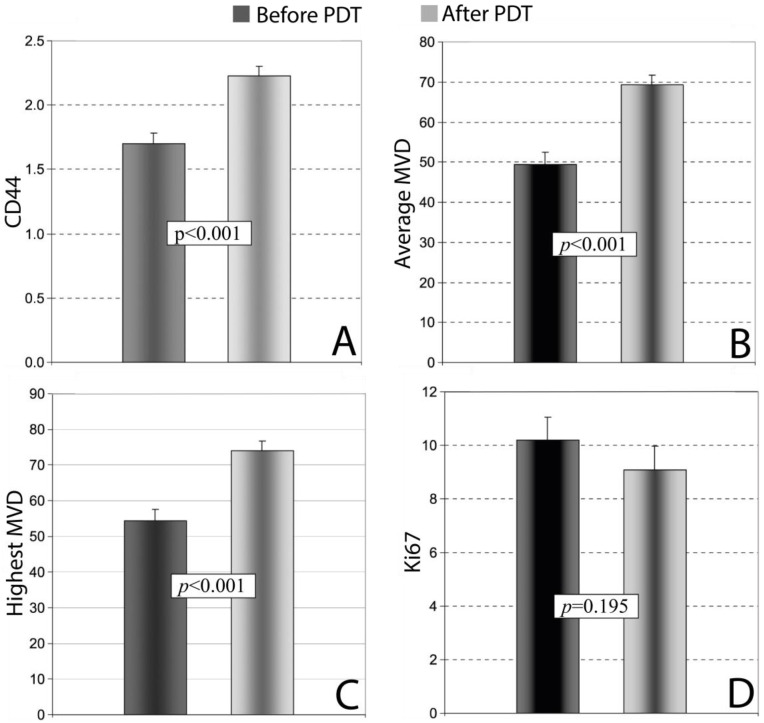
The analysis of expression levels in skin samples before and after PDT in women with VLS (*n* = 100): (**A**) CD44; (**B**) Average MVD; (**C**) Highest MDV; (**D**) Ki67. Abbreviations: PDT—photodynamic therapy; MVD—microvessel density [118].

The efficacy of PDT in the treatment of VLS was investigated using a immunohistochemical analysis [118]. The study focused on molecular markers of vascularization (CD34), nerve function, myelin basic protein (MBP), keratinocyte function (CD44), and the proliferation index (Ki67) in patients receiving PDT. A cohort of 100 patients with VLS was included, and skin biopsies were performed both before and after treatment, with analyses performed using antibodies against CD34, CD44, MBP, and Ki67. PDT demonstrated high efficacy in the treatment of VLS; in post-treatment biopsies, VLS persistence was confirmed in 39 (39%) women, while in the remaining 61 (61%) women, no histological signs of VLS were detected (*p* ≥ 0.001). Significant increases in the expression levels of CD44, CD34, and MBP were observed in histological images, which correlated with either partial or complete remission of both objective and subjective clinical symptoms (Figure 6). The average expression of CD44 changed in 32 (32%) women after PDT (*p* ≥ 0.001) (Figure 6A). A significant statistical difference was also found in the average and highest microvessel density (MVD) rates, as measured using a monoclonal anti-CD34 antibody, before and after therapy; the average MVD changed in 40% of cases (*p* ≥ 0.001), while the highest MVD changed in 36 (36%) cases (*p* ≥ 0.001) (Figure 6B,C). The positive expression of MBP increased significantly in the treated areas after PDT (by 138%, *p* ≥ 0.001). In contrast, the staining intensity rates for Ki67 did not exhibit any significant difference before and after PDT (*p* ≥ 0.05), which may indicate that the treatment is safe and does not lead to uncontrolled pathological cell proliferation (Figure 6D).

Spearman’s rank correlation test revealed a significant positive correlation between the highest and average MVD rates (R = 0.24, *p* ≥ 0.05), as well as between the highest MVD and Ki67 (R = 0.25, *p* ≥ 0.05), while a significant negative correlation was observed between average the MVD and CD44 (R = −0.25, *p* ≥ 0.05) [118].

The efficacy of PDT was further evaluated in a separate group of 102 patients with VLS [121]. The study demonstrated that PDT produced a positive therapeutic effect in 87.25% of patients, manifesting as complete or partial clinical remission. The most pronounced vulvoscopy response was characterized by a reduction in subepithelial ecchymoses and telangiectasias (78.95%), as well as a reduction in erosions and fissures (70.97%). Partial remission of lichenoid changes accompanied by hyperkeratosis was observed in 51.61% of cases, while the least pronounced response was seen in the reduction of atrophic lesions (an improvement in 37.36% of cases). The high clinical efficacy and safety of 5-ALA-PDT were shown in 30 VLS patients refractory to standard therapies. Six months post-treatment, all exhibited significant improvement; the initial hypopigmentation and atrophy resolved, and hyperkeratosis or sclerosis (present in 7 and 5 patients, respectively) diminished. The lesion size fell below 30% of the vulvar area in every patient (previously ≥60% in 21, ~30% in 7, <30% in 2), with 25 patients showing a marked reduction and 5 showing complete clearance. Histologically, the epidermal thickness decreased and dermal inflammation improved. Clinically, pruritus vanished in 27 patients and decreased from severe to moderate in the remaining three. The efficacy and safety of 5-ALA-PDT were first assessed in seven children with VLS [133]. After three sessions, the lesion size and hypopigmentation improved markedly, although ecchymosis and excoriation remained unchanged. All seven patients’ pruritus and six patients’ burning pain decreased significantly; two achieved complete pruritus remission and three complete pain remission. Symptom relief also occurred faster in children than reported in adults.

Ten VLS patients unresponsive to conventional treatment underwent three 5-ALA-PDT sessions [123]. Standardized digital photographs of the affected areas were taken before each session and at follow-up visits; no other therapies were administered. Quality of life was assessed via the DLQI. After treatment, pruritus fully resolved in nine patients and decreased from severe to mild in one. All patients showed significant lesion reductions; before treatment, lesions covered >60% of the vulvar surface in seven patients and 30–60% in three, while by the six-month follow-up point, the affected area had diminished in every case. The DLQI scores improved for all.

A retrospective analysis of 36 women with VLS refractory to conventional therapy [132] recorded objective signs, symptoms, and DLQI scores before and six months after 5-ALA-PDT. Complete remission of pruritus occurred in 24 (66.7%) patients, and the pruritus severity was reduced from severe to mild in 10 (27.8%) and persisted in 2 (5.6%). Complete remission of pain was seen in 16 (44.4%), and the pain was reduced to mild in 9 (25%) and remained intense in 2 (5.6%). The composite clinical scores fell from 5.31 ± 1.67 pretreatment to 3.67 ± 1.71 post-treatment. All patients reported improved DLQI scores.

Local PDT proved safe for gynecological disorders, with no adverse effects on fertility; all ten treated patients later delivered healthy, full-term children [81]. The cohort included two with squamous hyperplasia, two with vulvar sclerotic–atrophic lichen sclerosus (VLS), one with acuminate CCA, one with VIN I, two with CIN III, and two with CIN I. Following PDT, both the CIN I and CIN III lesions, as well as HPV-16, became undetectable. The VLS patients achieved complete remission after ten sessions. A treatment-resistant case of vulvar–vaginal CCA with HPV-16 also showed complete recovery. The histopathology confirmed complete remission in the VIN I–flat CCA patient. No cases of infertility were reported. All patients had healthy births; two delivered two children each, and five underwent cesarean sections.

PDT effectively improves most symptoms, clinical signs, and quality of life in early-stage VLS, with the benefits lasting at least three months but often waning by six months. In a retrospective study of 13 VLS patients [124], PDT achieved a 92.31% response rate, with lesions recurring in two patients at six months. The subjective, objective, and dermatological quality-of-life scores significantly respectively improved from 11.4, 4.3, and 13.4 at baseline to 4.9, 2.0, and 5.9 post-treatment (*p* < 0.05).

In 65 VLS patients [134], early-stage treatment (*n* = 44) achieved higher six-month efficacy rates than late-stage treatment (*n* = 21: 90.9% versus 76.2% (*p* < 0.05)), assessed using Cattaneo, DLQI, and FSFI methods. The early-stage patients showed significant reductions in pruritus, skin elasticity, depigmentation, and lesion extent (all *p* < 0.05), whereas in late-stage only pruritus, the depigmentation and lesion extents improved significantly (skin elasticity *p* = 0.0625). The FSFI scores rose in early-stage (median 17.45 → 21.10, *p* < 0.05) but not late-stage (10.55 → 10.00, *p* = 0.1865) patients. The DLQI scores improved in both groups (median early 7 → 4; median late 18 → 15; both *p* < 0.05).

Clinical and dermoscopic assessments in 24 VLS patients showed marked improvements after 5-ALA-PDT [128]. The lesion size, depigmentation, pruritus, and burning pain all decreased significantly after three sessions—with complete symptom remission in some—and continued to improve by the sixth session. Dermoscopically, the bright white or white-yellow amorphous areas diminished and vascular patterns increased after three treatments, with further favorable changes after six.

A two-year prospective controlled study of 100 VLS patients evaluated PDT’s immunomodulatory effects [120]. Group I (*n* = 60) had no autoimmune disease and group II (*n* = 40) had disorders (thyroid disease, vitiligo, arthritis). Pre-PDT, 60% exhibited severe symptoms. After ten sessions, 51% achieved complete symptom remission, 41% had a partial response, and 8% experienced persistent or worsened symptoms. In group II, 57% were antinuclear antibodies (ANA) positive pre-PDT, with the mean ANA levels dropping from 261.74 to 123.20 IU/mL post-treatment.

A retrospective analysis of 42 VLS patients unresponsive to topical glucocorticoids evaluated 5-ALA-PDT’s efficacy, recurrence, and side effects [131]. One year post-treatment, 64.3% (27/42) improved, 19.1% (8/42) showed partial improvement, and 4.8% (2/42) saw no change; recurrence occurred in 11.9% (5/42). The treatment efficacy rates showed no significant association with menopause, parity, weight, disease duration, or treatment timing, although patients with severe pruritus and skin atrophy had lower response rates. Furthermore, 5-ALA-PDT demonstrated low recurrence rates and minimal side effects while alleviating pruritus, enhancing elasticity and pigmentation, and reducing lesion sizes.

In 73 VLS patients [129], 17 HPV-positive cases (mostly HPV 16) were assessed using the Numeric Rating Scale (NRS) at 1, 3, 6, 12, and 24 months post-PDT. The numbers of PDT cycles required did not differ significantly between the HPV-positive and HPV-negative groups, including those needing three sessions. The remission durations showed no statistical difference, although the HPV-negative patients had a longer mean remission length (14 ± 9 vs. 11 ± 9 months).

Green light (495–570 nm) may be less painful and more effective than red light (630–635 nm) in PDT for VLS [135]. A study of 11 women with chronic VLS (1.5–4 years) included three PDT sessions at two-week intervals [122]. All five patients with vulvar erosions (100%) showed significant improvements, with complete resolution within two months. However, recurrences occurred in one patient after four months and two after six months, accompanied by burning. The pruritus decreased in 9 patients (81.8%) within two months; one reported mild and another moderate symptoms. Eight (72.7%) remained symptom-free for four months, and seven (63.6%) for six months. No pain was reported during the three PDT sessions.

Combined holmium laser therapy and 5-ALA-PDT enhances the VLS treatment efficacy [125]. A case study in one VLS patient involved combined ALA-PDT and holmium laser therapy. The lesions were irradiated with red light, administered in three sessions at two-week intervals. One month after the third ALA-PDT session, holmium laser ablation was applied to the lesions. After necrotic tissue removal, a final ALA-PDT session targeted the residual lesions. The lesion size was reduced, the hyperkeratotic plaques thinned, and the PS fluorescence intensity decreased after three ALA-PDT sessions. Post-treatment, nearly all lesions resolved, especially the residual hyperkeratotic plaques, with no scarring or stenosis, and the PS fluorescence further declined. The pruritus and dysuria were alleviated.

**Figure 7:**
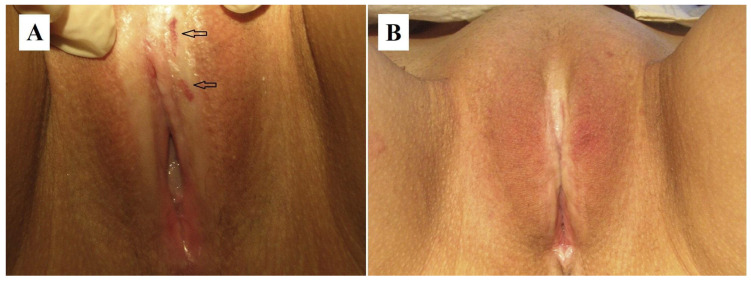
VLS lesions: (**A**) before treatment, where erosions are indicated by arrows; (**B**) after topical PDT, with complete remission of lesions. Abbreviations: VLS—vulvar lichen sclerosus; PDT—photodynamic therapy [119].

The combination of hormone therapy with local PDT is an effective treatment for VLS, particularly in resistant forms [119]. A patient with VLS and hypothyroidism participated in a study evaluating the simultaneous application of PDT and hormone therapy. A total of six PDT sessions were performed, resulting in complete symptom remission and mucosal recovery (Figure 7).

A study compared 4 PDT sessions with 10% 5-ALA (3 h accumulation) every two weeks (*n* = 20) versus daily 0.05% clobetasol propionate administration for 8 weeks (*n* = 20) in 40 VLS patients [117]. Both groups improved, although 5-ALA-PDT yielded longer-lasting effects and higher complete recovery (CR) rates. The CR rate was higher with PDT (14/20 vs. 7/20; *p* < 0.05, χ^2^ = 4.912). At 1 month post-PDT, only 1 patient relapsed versus full relapse (7/7) in the clobetasol group.

High-frequency ultrasound (HFUS) allows objective, quantitative monitoring of VLS treatment [130]. In a study of 31 patients with refractory VLS, both the subjective symptoms (itching, burning) and objective severity (lesion size, hypopigmentation) improved after ALA-PDT. Of 31 patients, 30 (96.8%) reported pretreatment itching or burning. The symptom severity declined significantly after the first ALA-PDT course, with a further reduction after the second, while 9/30 (30.0%) patients became symptom-free after the second course. The HFUS-measured hypoechoic dermal band (HDB) thickness decreased sequentially with ALA-PDT, correlating with a reduced histopathological inflammation depth (rs = 0.496, *p* = 0.005). The median HDB thickness dropped from 0.253 (0.193–0.367) mm at baseline to 0.203 (0.178–0.260) mm after the first course and 0.170 (0.136–0.213) mm after the second. The collagen homogenization depth and inflammatory infiltration also decreased significantly post-treatment, from 0.168 (0.113–0.242) mm to 0.087 (0–0.131) mm and 0.312 (0.269–0.399) mm to 0.258 (0.192–0.355) mm, respectively.

#### 3.2.2. MAL

MAL-PDT offers a number of advantages for the treatment of VLS in women, including high efficacy with minimal risk of damage to healthy tissue and the occurrence of side effects such as scarring and hyperpigmentation, which is especially important in delicate areas. The procedure also avoids surgical intervention, providing an aesthetically acceptable result without a long recovery period. In addition, MAL-PDT can be used repeatedly, making it suitable for chronic cases. The use of MAL as PS for VLS PDT alleviates the symptoms of the disease and improves the appearance of the affected skin areas, which has a positive effect on the psychological well-being of female patients, prevents the progression of VLS, and improves the long-term prognosis [136].

In the course of the literature review, one article was studied that investigated the efficacy and safety of MAL-FDT for the treatment of VLS in eight patients [136] (Table 6).

Topical MAL (160 mg/g) was applied for 3 h, followed by irradiation with 630 nm red light (70 mW/cm^2^, 37 J/cm^2^ over 9 min 45 s). The patients underwent 1–3 PDT sessions at 6–12-month intervals, with clinical follow-up at 4, 8, and 12 weeks, then every 3 months. The pretreatment and follow-up assessments included VAS for itching, pain, burning, and dyspareunia, along with DLQI. The biopsy and histology confirmed the efficacy. All patients showed improved symptoms and quality of life. However, no quantitative data on the complete response (CR) rates or HPV eradication were provided [136].

PDT using 5-ALA and MAL has been widely used to treat various diseases including cervical tumors and VLS. Although the mechanisms of activation of the antitumor and immune response are similar, differences in tissue characteristics and the nature of the diseases result in unique aspects of each PS.

5-ALA-based PDT targets cervical tumors by inducing ROS production and releasing DAMPs (HMGB1, HSP70), which recruit dendritic cells and Langerhans cells (LCs) to present tumor antigens to T lymphocytes, activating antitumor immunity [137]. Due to its hydrophilicity, 5-ALA is selectively metabolized into PpIX in proliferating tumor cells, enabling targeted destruction while sparing healthy tissue. PDT-induced inflammation enhances the infiltration of macrophages and CD8+ T cells, amplifying systemic antitumor responses.

In treating vulvar sclerosing lichen planus with MAL, the primary aim is to reduce chronic inflammation and prevent malignant progression. Compared to 5-ALA, MAL’s higher lipophilicity enables deeper penetration into damaged epithelial tissues. PDT’s activation of MAL triggers the release of proinflammatory cytokines (IL-6, TNF-α), promoting tissue remodeling and the suppression of chronic inflammation. Unlike 5-ALA, MAL does not target malignant cells but restores the epithelial microenvironment and limits fibrosis. The antitumor immune responses are minimal due to the absence of malignant cells and associated antigens for T cell activation [138].

Thus, in the treatment of cervical tumors, 5-ALA activates both innate and adaptive immunity by destroying tumor cells and releasing antigens. In the case of vulvar sclerosing lichen planus, MAL acts predominantly through the modulation of local inflammation and tissue remodeling, and the systemic antitumor effect is minimally expressed. These differences reflect the importance of an individual approach to the choice of PS depending on the nature of the disease and the goals of therapy.

The overall analysis of the treatment results in VLS PDT showed that a CR of more than 90% was achieved in 9 of 20 studies using 5-ALA. HPV eradication outcome information was reported in only 2 of 20 articles for 5-ALA and did not reach more than 90%. The authors of the article in which MAL was used did not provide quantitative data on the results of CR and HPV elimination.

The heterogeneity of the PDT treatment results (less than 90% CR) in patients with VLS using 5-ALA and MAL may be related to the following factors:

1. Differences in PDT protocols:
  a PS concentration (5–20% for 5-ALA; 160 mg/g for MAL);
  b Exposure time (2–5 h);
  c Parameters of light exposure (power density from 40 to 204 mW/cm^2^, energy density from 37 to 150 J/cm^2^);
  d Number of treatment sessions (from 1 to 10).
2. Patient characteristics:
  a Disease stage (early or late);
  b Immune response;
  c Associated diseases (autoimmune diseases or oncologic processes).
3. Methodological factors:
  a Diagnostic methods and performance criteria (biopsy, histology, cytology, colposcopy, vulvoscopy, HPV PCR, etc.);
  b Duration of patient follow-up (3 months to several years);
  c Methods of therapy dosimetry (presence or absence of fluorescence diagnostics).

Despite the variety of PSs used in clinical practice for cervical and vulvar PDT, their direct comparison within a single study is difficult due to differences in design, inclusion criteria, and treatment protocols in the analyzed publications. Nevertheless, based on the data collected, it is possible to highlight the features that determine the applicability of each PS in different clinical conditions. Thus, 5-ALA and its derivatives HAL and MAL demonstrate high safety, minimal invasiveness, and an organ-preserving effect, which makes them particularly suitable for the treatment of CIN I–II in women of reproductive age. HAL and MAL allow deeper penetration and selective accumulation in affected tissues, reducing the impact on the healthy epithelium. At the same time, Ce6, which has a pronounced ability to accumulate mainly in blood vessels and induce an immune response, has demonstrated high efficacy in the treatment of more severe lesions (CIN III, CIS, MIC). Thus, 5-ALA and its derivatives are preferable for superficial and early lesions, whereas Ce6 is a more justified choice for extensive or deeply localized processes. The inclusion of such comparisons in clinical protocols may contribute to the personalization of PDT and increase its efficacy.

The PDT parameters presented in Table 1, Table 2, Table 3, Table 4, Table 5 and Table 6 were systematically collected and analyzed to identify patterns that determine the clinical efficacy and safety of the treatment of cervical and vulvar lesions. A synthesis of the presented results yielded several practical conclusions. In the case of cervical lesions, the best lesion and HPV regression rates were achieved using Ce6 at a concentration of 0.8–2.5 mg/kg and exposure to radiation with a wavelength range of 660–670 nm, a power density range of 200–300 mW/cm^2^, and a light dose range of 100–400 J/cm^2^. For vulvar lesions, 5-ALA demonstrated the greatest efficacy, especially at a concentrations of 10–20%, a radiation wavelength range of 630–635 nm, a power density range of 80–200 mW/cm^2^, and a light dose range of 60–150 J/cm^2^. In the case of HPV-associated lesions, the utilization of Ce6 is recommended due to the high probability of complete HPV eradication and sustained regression of lesions. Concurrently, in the therapeutic management of non-viral VLS, 5-ALA continues to be recognized as the most efficacious PS.

## 4. Future Perspectives

PDT is a promising, minimally invasive method for treating CIN I–III, CIS, CCA, and VLS, showing high antitumor and antiviral efficacy [77,102,106,131,139,140]. It offers advantages such as high selectivity, few side effects, and compatibility with other therapies. However, its clinical application faces challenges, including limited light penetration, tumor heterogeneity, uneven PS distribution, low PS selectivity, and tumor hypoxia, all reducing its efficacy. Additionally, the PS often accumulates in TAMs, whose presence rates vary from 5% to 60% depending on the tumor type and stage, significantly affecting the treatment outcomes. Future research should focus on personalizing the PDT parameters, developing better PS and delivery methods, and using noninvasive techniques such as time-resolved spectroscopy to assess the macrophage polarization [141,142]. Understanding the molecular and cellular mechanisms can help modulate immune responses via macrophages and LCs, improving the safety and effectiveness of HPV and tumor treatments.

The clinically available PSs are mainly activated by light in the 400–700 nm range [143,144,145,146,147,148], which limits the tissue penetration to 0.5–2.5 mm, hindering the treatment of deep tumors. The use of phototheranostics in the near-infrared (NIR) range—specifically NIR-I (700–1000 nm) and NIR-II (1000–1700 nm)—offer advantages such as deeper penetration, better imaging contrast, higher safety, lower phototoxicity, and low autofluorescence [149,150,151,152,153,154,155]. However, the clinical use of NIR-based PDT is limited because wavelengths above 700 nm are less efficient at generating ROS, which are crucial for PDT;s efficacy [111,156,157,158]. This reduced ROS production stems from decreased oxygen excitation efficiency, restricting the therapeutic effectiveness of NIR light despite its other benefits.

Numerous new PSs capable of absorbing NIR radiation have been developed, including nanoparticle-based PSs, indocyanine green (ICG), BODIPYs, Keio Fluors, Aza-BODIPY, chlorins, phthalocyanines, and cyanine dyes [159,160,161]. However, despite their potential, only a few have entered clinical trials, and very few are currently used in cancer treatment [159]. As a result, NIR-range phototheranostics has not yet become a clinical standard in oncology, although ongoing research and technological progress may facilitate its adoption in the near future.

Modern NIR-absorbing dyes, such as BODIPY-based photosensitizers (PSs), offer deep tissue penetration and enhanced photophysical properties, improving the therapeutic efficacy. BODIPY dyes are especially promising due to their high fluorescence, photochemical stability, and tunable absorption and emission wavelengths through structural modifications [162]. While type II PDT relies on singlet oxygen generation, a key factor for its effectiveness [163], the type I mechanisms involving radical formation can enhance PDT’s activity under low-oxygen conditions [164,165]. In vitro studies using Henrietta Lacks (HeLa) treated with a BODIPY-based PS at concentrations of 0.05, 0.50, and 1.25 μM showed an approximately twofold ROS increase after light exposure, measured via the National Benchmark Test (NBT) [166]. These findings suggest that BODIPY operates via both type I and type II mechanisms, offering complementary advantages that may improve the PDT outcomes [167].

A major challenge in PDT is the non-specific distribution of the PS, which can damage healthy tissues. To address this, various targeted delivery systems are being developed, particularly nanoparticle-based carriers such as gold nanoparticles, titanium dioxide nanoparticles, and quantum dots, which enhance the light absorption and ROS generation due to their plasmonic and semiconductor properties [168,169,170,171,172,173,174,175,176]. These nanoparticles also allow for improved biodistribution and reduced side effects through functionalization and targeted delivery to cancer cells [168]. Liposomes are among the most studied PS delivery systems for cervical cancer, offering biocompatibility, controlled release, and protection of the PS from degradation [169,177,178,179]. Liposomes can be modified for the specific targeting of certain cancer cell types, thereby increasing the therapeutic efficacy [177]. The use of ethosomal formulations is another approach that increases the penetration of the PS into deep tissues [180]. In clinical studies, chitosan-based AlClPc nanoparticles in gel form showed promising results in treating CIN I and II in 12 patients, whereby 91.7% showed favorable outcomes with no observed toxicity and 91.7% had negative Papanicolaou smear results post-treatment [181]. Hypericin (HYP) is a promising PS, although the data on its use in cervical cancer remain limited [182]. The majority of the available data were obtained from studies conducted on the HeLa cell line [183,184,185,186]. In one study [187], HYP delivered via P123 Pluronic^®^ micelles demonstrated high efficacy against cervical cancer cells without harming HaCaT cells, acting primarily through a type II PDT mechanism, while also inhibiting cancer cell migration and invasion. Integrating various therapeutic modalities into a unified system may help overcome the limitations inherent to traditional treatment methods and significantly improve outcomes in oncology [182]. It has been shown that polymeric PPHE nanoparticles with enhanced localization capabilities in the body improved the efficacy of combined PDT–CAP therapy in CaSki cells [188]. PDT and CAP halted the growth of cervical cancer cells by increasing the ROS levels and triggering apoptosis, as confirmed by a 3D cell culture model, which demonstrated the therapeutic potential of PPHE-based combination therapy.

Methylene blue (MB) is a widely used PS in PDT. It exhibits high mitochondrial affinity and induces mitochondria-dependent apoptotic cell death in HeLa cells [189]. MB also alters the polarization of macrophage, potentially reducing their antitumor activity and modifying the tumor microenvironment [190]. Additionally, MB restores cellular respiration [191,192,193,194], increasing the intracellular oxygen levels, which may lead to reduced cancer cell malignancy and enhanced healthy cell survival [195]. MB is often combined with nanoparticles for improved cancer treatment [196,197,198]. For example, “core–shell” anisotropic ferrite polymer nanoparticles have been developed for cervical cancer photodynamic ablation [199]. MB-CuFe, synthesized via hydrothermal reaction, enhances the effectiveness of phototherapy by acting as a Fenton catalyst to generate ROS, offering the potential for MRI and photothermal therapy due to its high conversion efficiency under deep red light [199]. Nanoparticles serve as effective PS carriers, improving the localization precision and drug concentration rates at tumor sites [200]. For instance, PpIX-loaded nanoparticles show higher therapeutic efficacy and reduced toxicity compared to free PS [200].

Tumor hypoxia is a major challenge in PDT, as it limits the availability of the oxygen needed for ROS generation and reduces the treatment efficacy [64]. To address this, strategies targeting cancer cell mitochondria—key regulators of apoptosis and metabolism—are being explored, showing promise due to the mitochondria’s susceptibility to oxidative damage and hyperthermia. Mitochondria-targeting PSs can enhance the tumor response under hypoxic conditions [201,202]. ICG derivatives such as IR-780 are suitable for PDT due to their NIR absorption; however, ICG faces limitations such related to photobleaching, low photothermal efficiency, and aggregation in water, which reduces its stability and effectiveness [203,204,205,206]. After intravenous administration, ICG binds to plasma proteins (e.g., albumin, α-1 lipoproteins) and is rapidly cleared, with a half-life of 2–4 min [207,208]. Without delivery systems such as nanoparticles and liposomes, its tumor specificity is low [209,210]. Its encapsulation in nanocarriers such as hyaluronic acid (HA) improves its targeted delivery to CD44-overexpressing cancer cells [211]. A new ICG derivative, IR-Pyr, accumulates in mitochondria and shows improved photostability [212]. In vitro and in vivo studies have confirmed that the IR-Pyr–HA complex enhances the tumor-specific delivery and PDT efficacy [213]. In one study [214], a nanobiomimetic system using M-HMnO_2_@ICG nanoparticles was developed for improved tumor oxygenation and to respond to the tumor microenvironment, enabling effective cervical cancer PDT with minimal side effects via single laser irradiation. These nanopreparations evade immune clearance processes, accumulate more in tumors, and reduce the systemic toxicity, enhancing the antitumor effectiveness [214].

A promising approach is Cherenkov radiation-induced PDT [215]. Unlike conventional PDT, which uses external light sources, Cherenkov PDT generates light endogenously within tissues using radioactive isotopes. This method offers two key advantages:

1. Higher signal-to-noise ratios in optical imaging due to unidirectional light propagation from the radionuclide to the detector [216];
2. Depth-independent photodynamic effects, as long as the photosensitizer and Cherenkov emitter are closely localized [216].

Despite its advantages, Cherenkov PDT faces challenges such as a limited photon output, a narrow luminescence spectrum, and interference from ambient light, which hinder its broader use [217]. Currently, 5-ALA-induced PpIX and 18F-FDG are most commonly used as the photosensitizer and radiopharmaceutical, respectively, due to their active uptake by cancer cells [218]. In vitro and in vivo studies [219] on ES2 ovarian cancer cells showed that Ce6 and Verteporfin (VP), when combined with 18F-FDG, reduced the cancer cell viability rates by 31.5% and 21.4%, respectively [219]. VP was more effective for singlet oxygen generation and extended median survival from 14.5 to 18.5 days in a mouse model [219].

PDT is a promising organ-sparing approach for treating gynecologic cancers, offering high selectivity and the ability to trigger antitumor immune responses. The research highlights the role of LCs in capturing tumor antigens released after PDT-induced cell death [137]. DAMPs such as HSP70 and HMGB1 stimulate LC migration to lymph nodes, where they activate T lymphocytes, driving adaptive immunity [220].

Enhancing Langerhans cell (LC) activation involves using nanomaterials for targeted photosensitizer delivery to tissues with high LC densities. For instance, ligand-modified liposomes improve the LC–antigen interaction, strengthening the antitumor immune response.

Developing NIR-activated PS is vital for advancing PDT. Dyes such as BODIPY and phthalocyanines have high singlet oxygen quantum efficiency rates, improving their efficacy in hypoxic tumors [111], and they can be structurally modified for targeted tumor delivery, reducing the side effects [115].

Nanomaterials enhance PDT’s selectivity by improving the stability and biodistribution of the PS. Liposomes, ethosomes, and gold or titanium dioxide nanoparticles enable targeted delivery. For example, Pluronic^®^ micelles increase the accumulation of the PS in tumor cells, boosting the ROS generation and antitumor immune responses.

Clinical studies show that PDT using nanomaterials and novel photosensitizers effectively treats cervical and vulvar tumors, regardless of the HPV status. Combining PDT with chemotherapy or immunotherapy boosts the systemic antitumor response and lowers the recurrence risk. Its integration with imaging enables personalized treatment and real-time monitoring of the PS distribution, enhancing the overall efficacy.

Finally, integrating advanced real-time monitoring tools such as time-resolved fluorescence spectroscopy and multimodal imaging into clinical PDT protocols can enable personalized dosimetry, allowing clinicians to dynamically adapt their treatments to the tumor response and PS distribution, thereby improving the efficacy and safety [141,142].

Future progress in PDT for gynecological neoplasms relies on the following factors:

- Personalized treatment protocols adapted to tumor biology and patient-specific factors, including hypoxia and immune status;
- Next-generation NIR-activated PSs with improved ROS generation and selectivity;
- Innovative nanotechnology-based PS delivery systems to improve tumor targeting and tissue penetration;
- Strategies to overcome tumor hypoxia, including mitochondrial targeting and oxygen supplementation;
- The exploitation of PDT-induced antitumor immunity through its combination with immunotherapies and the targeted activation of innate immune cells;
- The development of noninvasive real-time imaging and dosimetry tools to monitor the PS localization and treatment progress.

Addressing these priorities will be crucial to realize the full potential of PDT as a safe, effective, and organ-preserving treatment for cervical and vulvar neoplasms.

This systematic review provides a comprehensive evaluation of the current clinical and experimental applications of PDT in the treatment of cervical and vulvar neoplasms. The collected evidence highlights PDT as a promising organ-preserving therapeutic option, with demonstrated efficacy in eradicating HPV-associated lesions such as CIN I–III, CIS, and VLS, and with a favorable safety profile. One of the strengths of the review is the detailed comparison of various photosensitizers, light sources, dosimetric parameters, and clinical outcomes, allowing for a clearer understanding of how the treatment protocols can be optimized for better efficacy and reduced side effects.

However, when interpreting the obtained results, a number of methodological limitations of the studies included in the review should be taken into account. A significant number of the studies had a limited sample size (in some cases less than 30 patients), which reduces the statistical power and makes it difficult to generalize the results. There were also variations in PDT protocols, including differences in PS concentrations, parameters of irradiation, diagnostic methods, and criteria for assessing efficacy. In a number of publications, the period of clinical follow-up did not exceed 3–6 months, which does not allow a full assessment of the recurrence rate and long-term effects of therapy. It should be noted that most of the studies included in the review were conducted in Russia and China. Differences in clinical guidelines, approaches to diagnosis, and the implementation of PDT may affect the reproducibility of the results in other countries. However, our analysis focused primarily on the comparison of specific therapy parameters (type of photosensitizer and its concentration, irradiation parameters, duration of exposure, etc.) and their association with the clinical outcomes, which allowed us to identify universal patterns potentially relevant to different healthcare systems. Another factor to consider is the potential risk of publication bias; most of the selected studies reported predominantly positive results, while negative or ineffective cases may have remained unpublished. Taken together, these aspects limit rigorous comparisons and require caution in interpreting the summarized data. In future studies, it is desirable to use standardized protocols, larger patient cohorts, and longer follow-up periods to improve the reliability and reproducibility of the results.

Given the preliminary nature of the majority of studies included in the review, which often featured limited patient numbers and varying methodologies, there is a need for a conceptual model that can integrate the presented data and offer a holistic understanding of the clinical efficacy of PDT in gynecologic oncology. A systematic analysis of the available literature yielded our working hypothesis that the long-term clinical effect of PDT in HPV-associated lesions is determined not only by its direct cytotoxic action but also by the ability of the therapy to induce a localized, controlled inflammatory response that activates antigen-presenting cells (particularly Langerhans cells) and triggers an adaptive immune response aimed at the elimination of HPV-infected cells. This hypothesis was corroborated by the observed correlations between the irradiation parameters, type of photosensitizer, degree of T cell activation, and clinical remission rates. Consequently, the immunomodulatory capacity of PDT should be regarded as a pivotal element in therapeutic interventions. This theoretical basis may serve as a foundation for subsequent clinical and immunological studies aimed at optimizing personalized PDT protocols.

Future research studies should prioritize the development of unified protocols for PDT administration in gynecological oncology, along with consistent documentation of the treatment parameters and outcomes. Long-term prospective studies are needed to evaluate the durability of the clinical response, recurrence risks, and patient quality of life. Finally, incorporating real-time dosimetry and imaging techniques may help refine the treatment’s precision and improve the therapeutic outcomes.

## 5. Conclusions

Advanced research studies of the molecular and cellular mechanisms of PDT and the development of improved PS delivery methods will help enhance cervical cancer treatments by increasing their safety and efficacy. These advancements will enable personalized PDT parameters tailored to patient tumors. The combined effect of these improvements will also reduce the risk of potential side effects. A deeper investigation into the mechanisms of immune system activation in response to photodynamic exposure may further amplify the antitumor effect by not only destroying tumor cells but also by stimulating immune cells to eliminate them, thereby significantly improving the patient survival rates.

## Figures and Tables

**Table 1:** Description of articles included in the review.

Reference Year Country	Localization HPV Type	Numbers of Patients	PS Concentration Accumulation Time, h	Wavelength, nm Power Density, mW/cm^2^ Energy Density, J/cm^2^ Exposure, Min	Repetition Rate Number of Courses	Diagnostics	HPV Outcomes	Lesion Outcomes
Wang [76] 2010 China	CIN II: 80% (4/5), CIN III: 20% (1/5) HPV 16: 80% (4/5), HPV 59: 20% (1/5)	5	5-ALA 118 mg/g 3–4	635 100–150 100 20–28	Once every 1–2 weeks 2–4	Biopsy, histology, colposcopy, HPV DNA testing	80% (4/5)—negative, 20% (1/5)—positive	80% (4/5)—CR for 9 months after 3–4 courses, 20% (1/5)—PR for 6 months
Chen [77] 2011 China	CCA HR-HPV	48	5-ALA 20% 3	630 100 100 Not	Once every week 3	Biopsy, histology, electron microscopy, HPV immunohistochemistry stain	Not	95.8% (46/48)—CR after 3 courses
Wang [78] 2012 China	CCA HPV 16: 1.8% (1/56), HPV 18: 1.8% (1/56), HPV 6: 28.6% (16/56), HPV 11: 48.2% (27/56), HPV 16 and HPV 6: 5.4% (3/56), HPV 16 and HPV 11: 10.7% (6/56), HPV 18 and HPV 6: 3.6% (2/56), HPV18 and HPV 11: 1.8% (1/56)	56	5-ALA 10% 4	635 100 100 Not	Once every 2 weeks 1–4	Colposcopy, histology, HPV genotyping	83.9% (47/56)—negative after 4 courses	98.2% (55/56)—CR after 4 courses
Fu [79] 2016 China	CIN I HR-HPV	Total: 76 (treatment group—39; control group—37)	5-ALA 10% 3	635 Not 100 Not	Once every 2 week 3	Hybrid capture HPV DNA assay, TCT, colposcopy	Treatment group: 64.10% (25/39); control group at 3 months: 4.32% (9/37); treatment group: 76.92% (30/39) at 9 months; control group: 32.40% (12/37) at 9 months	Treatment group: 81.81% (9/11) at 3 months; control group: 12.50% (1/8) at 3 months; treatment group: 90.90% (10/11) at 9 months; control group: 25.00% (2/8) at 9 months; treatment group: 83.3% (5/6)—CR at 9 months; control group: 0% (0/5)—CR at 9 months
Liu [80] 2016 China	CIN I	Total: 110 (treatment group—55; control group—55)	5-ALA 20% 3	632.8 Not 100 40–50	Once every 7–10 days 4	Colposcopy, TCT, HPV DNA	Treatment group, total: 92.73% (51/55) (81.81% (45/55) at 6 months and 10.91% (6/55) at 9 months); control group, total: 60.0% (33/55) (52.73% (29/55) at 6 months and 7.27% (4/55) at 9 months)	Treatment group, total: 98.18% (54/55) (92.72 (51/55)—cure, 5.45% (3/55)—improvement, 1.18% (1/55)—invalid); control group, total: 70.91% (39/55)—cure, 10.91% (6/55)—improvement, 29.09% (16/55)—invalid
Maździarz [81] 2019 Poland	SCC: 2, VLS: 2, genital warts: 1, VIN I: 1, CIN III: 2, CIN I: 2 HPV 16: 5, HPV 31: 1, HPV 42: 1	10	5-ALA 5% 3	590–760 204 120 10	Once every week 10	Colposcopy, vulvoscopy, biopsy, histology, HPV DNA testing	17% (1/6)—negative	90% (9/10)—CR; 10% (1/10)—PR
Ran [82] 2021 China	CIN I: 61.11% (33/54); CIN III: 18.52% (10/54); Simple HR-HPV: 20.37% (11/54) HR-HPV	54	5-ALA 20% 3	635 Not Cervix: 100; Cervical canal: 350 Not	Once every week 6	Fluorescence quantitative PCR for HPV detection, colposcopy, colposcopy-guided biopsy for CIN detection	CIN I: 63.64% (14/22) after 6 courses; CIN III: 50% (4/8) after 6 courses; simple HR-HPV: 71.43% (5/7) after 6 courses	CIN I: 69.57% (16/23) at 10 weeks; CIN III: 75% (6/8) at 10 weeks; simple HR-HPV: 80% (4/5) at 10 weeks
Wu [83] 2021 China	CIN II HR-HPV 16, 18: 51.61% (16/31); Other 12 HR-HPV: 48.39% (15/31)	31	5-ALA 20% 4	635 Not 100 Not	Once every 1–2 weeks 3	Cytology, HPV genotyping, colposcopy-directed biopsy	70.97% (22/31)—CR for 6 months; 62.96% (17/27)—CR for 12 months; 29.03% (9/31); 37.04% (10/27)—CR for 12 months	Disappearance: 77.78% (21/27); regression: 14.81% (4/27); persistence: 7.41% (2/27)
Qu [84] 2022 China	CIN III HR-HPV	96	5-ALA 20% 4	635 Not 100 20	Once every 1–2 weeks 6	Biopsy, histology, colposcopy, TCT, HPV typing	79.17% (76/96)—negative after 3 months	89.58% (86/96)—CR after 3 months
Hu [75] 2022 China	CIN II: 18; CIN III: 4 HR-HPV 16, 18, 31, 33, 45, 51, 52, 56, 58, 59, 66, 68, 82 (single type: 59.09% (13/22); two or more types: 40.91% (9/22))	22	5-ALA 20% 4	635 Not 100 4	Once every week 6	TCT, HPV DNA testing, HPV E6/E7 mRNA examination, colposcopy, biopsy, immunohistochemistry detection	54.55% (12/22)—negative after 3 months; 86.36% (19/22)—negative—after 6 months	81.82% (18/22)—CR after 3 months; 90.91% (20/22)—CR after 6 months
Bizoń [85] 2022 Poland	VLS, VIN, CIN, EIN Not	Total: 182 (group 1 (patients with VIN, CIN, EIN): 44; group 2 (patients with family cancer history): 51; group 3 (control): 87)	5-ALA 2 mg/mL 2	630 204 Not 10	Once every week 10	Vulvoscopy, questionnaire	Not	Vulvar changes: (group 1: 21.9%; group 2: 21.2%, group 3: 21.8%); itching: (group 1: 39.3%; group 2: 35.5%, group 3: 42.5%)

Abbreviations: HPV—Human Papillomavirus; CIN—Cervical Intraepithelial Neoplasia; CCA—Cervical Condylomata Acuminata; HR—High-Risk; SCC—Squamous Cervical Cancer; VLS—Vulvar Lichen Sclerosus; VIN—Vulvar Intraepithelial Neoplasia; EIN—Endometrial Intraepithelial Neoplasia; PS—Photosensitizer; 5-ALA—5-Aminolevulinic Acid; DNA—Deoxyribonucleic Acid; TCT—ThinPrep Cytology Test; PCR—Polymerase Chain Reaction; mRNA—Messenger Ribonucleic Acid; CR—Complete Response; PR—Partial Response. A subsequent analysis of the materials and methods of the 11 articles listed revealed a similar algorithm for patient preparation for PDT of CIN, cervical cancer, and CCA. Before PDT, patients’ cervical canal, cervix, and vagina were washed with sterile physiological solution, then a cotton pad or swab soaked with freshly prepared aqueous solution or gel containing 5–20% 5-ALA was applied to the lesion area for 2–4 h. The application of a gauze or cap over the cotton pad or swab was used to protect the exposed area from light.

**Table 2:** Description of articles included in the review (continuation).

Reference Year Country	Localization HPV Type	Numbers of Patients	PS Concentration Accumulation Time, h	Wavelength, nm Power Density, mW/cm^2^ Energy Density, J/cm^2^ Exposure, Min	Repetition Rate Number of Courses	Diagnostics	HPV Outcomes	Lesion Outcomes
Soergel [93] 2010 Germany	CIN I: 12% (3/25), CIN II: 36% (9/25), CIN III: 52% (13/25) HR-HPV	25	HAL, MAL HAL: 10 mM, HAL: 40 mM, MAL: 1.2 M 12	633 Not 100 17	Not 1–2	Biopsy, histology, immunohistology, colposcopy	Not	Total: 64% (16/25): 36% (9/25)—CR after 6 months, 28% (7/25)—PR after 6 months
Soergel [94] 2012 Germany	CIN I: 9% (8/92), CIN II: 25% (23/92), CIN III: 39% (36/92) HR-HPV: 84% (65/77)	92	HAL, MAL HAL: 10 mM, HAL: 40 mM, MAL: 1.2 M 3, 12	633 25–100 25–100 17	Once every 4 weeks 1–2	Biopsy, histology, colposcopy, cytology, HPV DNA testing	14/21 (67%) CR	Part 1, after 6 months: MAL 1.2 M, 3 h, 100 J/cm^2^: 50% (6/12); HAL 10.0 mM, 3 h, 100 J/cm^2^: 33% (4/12); HAL 40.0 mM, 3 h, 100 J/cm^2^: 46% (5/11); MAL/HAL, 3 h, 100 J/cm^2^: 43% (15/35); MAL 1.2 M, 12 h, 100 J/cm^2^: 18% (2/11); HAL 10.0 mM, 12 h, 100 J/cm^2^: 0% (0/11); HAL 40.0 mM, 12 h, 100 J/cm^2^: 20% (2/10); MAL/HAL, 12 h, 100 J/cm^2^: 13% (4/32); MAL 1.2 M, 3 + 12 h, 100 J/cm^2^: 35% (8/23); HAL 10.0 mM, 3 + 12 h, 100 J/cm^2^: 17% (4/23); HAL 40.0 mM, 3 + 12 h, 100 J/cm^2^: 33% (7/21); Part 2, after 6 months: HAL 40.0 mM, 3 h, 50 J/cm^2^: 33% (6/18); HAL 40.0 mM, 3 h, 25 J/cm^2^: 29% (2/7)
Hillemanns [95] 2014 Germany	CIN I HR-HPV: 43% (30/70)	Total: 70 (treatment group—47, control group—12, follow-up group—11)	HAL 100 mg 5	633 Not 50 17	Once every 4 weeks 1–2	Colposcopy, cytology, HPV testing	Treatment group: 73% (11/15)—negative; control + follow up group: 50% (5/10)	Treatment group: 57% (27/47)—CR after 6 months; control + follow up group: 25% (23/47)
Inada [96] 2014 Brazil	CIN I, CIN II Not	23	MAL 20 CIN I: 1, CIN II: 3	630 CIN I: 80, CIN II: 120 CIN I:100, CIN II: 150 21	Single treatment protocol 1	Colposcopy, Papanicolaou smear, biopsy, fluorescence imaging	Not	100% (23/23)—CR
Hillemanns [97] 2015 Germany	CIN I: 118, CIN II: 89, placebo: 55 HR-HPV 16, 18	262	HAL 0.2%, 1%, 5% 5	629 Not 100 276	Not 1–2	Biopsy, Papanicolaou test, HPV test	HAL 5%: 84% (16/19), HAL 1%: 48% (14/29), HAL 0.2%: 42% (8/19), Placebo: 38% (8/21)	HAL 5%: 95% (18/19), HAL 1%: 69% (20/29), HAL 0.2%: 63% (12/19), placebo: 57% (12/21)
Hass [90] 2017 Germany	CIN II HR-HPV 16	Total: 2 (PDT group: 1; placebo group: 1)	HAL 0.2% Not	Not Not Not Not	Not 2	Colposcopy, HPV testing, cytology, blood sampling	Not	Not

Abbreviations: HPV—Human Papillomavirus; CIN—Cervical Intraepithelial Neoplasia; HR—High-Risk; PS—Photosensitizer; HAL—Hexaminolevulinate; MAL—Methylaminolaevulinate; DNA—Deoxyribonucleic Acid; CR—Complete Response; PR—Partial Response. Based on the materials and methods of the analyzed studies, HAL and MAL were applied locally to the affected areas in the form of a solution, ointment, or suppository. The solution or ointment was applied to the affected area using a gynecological applicator or a needle-free syringe. Suppositories were administered intravaginally. A tampon was used to retain the solution, ointment, or suppository at the site of the lesion. For PDT, solutions containing 10 mM and 40 mM of HAL [93,94]; 0.2%, 1%, and 5% solutions or ointments of HAL [90,97]; or suppositories containing 100 mg of HAL [95] were used. In two studies, the concentration of MAL in the solution was 1.2 mM [93,94], and in one study [96] a 20% MAL solution was used. The application times for HAL and MAL in the cited studies varied from 1 to 12 h, depending on the severity of the lesion.

**Table 3:** Description of articles included in the review (continuation).

Reference Year Country	Localization HPV Type	Numbers of Patients	PS Concentration Accumulation Time, h	Wavelength, nm Power Density, mW/cm^2^ Energy Density, J/cm^2^ Exposure, Min	Repetition Rate Number of Courses	Diagnostics	HPV Outcomes	Lesion Outcomes
Istomin [101] 2010 Belarus	CIN II: 24, CIN III: 88 HR-HPV: 78.6% (88/112)	112	Ce6 1.0–2.5 mg/kg 3–4	670 >200 100 20	Single treatment protocol 1	Biopsy, histology, bacteriological and cytological examinations, colposcopy, Lugol’s iodine test, a PCR analysis of HPV	53.4% (47/88)	92.8% (104/112)—CR; 2.7% (3/112)—PR
Alekseeva [102] 2020 Russia	CIN I: 2, CIN II: 2, CIN III: 5, CIS: 1 HPV 16, 18, 6, 11	10	Ce6 0.8–1.2 mg/kg 3	660 0.13–1.02 100–250 Not	Single treatment protocol 1	Video- and spectral-fluorescence diagnostics, biopsy, a PCR analysis of HPV	90% (9/10)—negative, 10% (1/10)—positive	90% (9/10)—CR, 10% (1/10)—PR
Ivanova [103] 2020 Russia	CIS HR-HPV 16, 18, 31,33, 35, 45, 56: 82% (37/45)	Total: 45 (group 1, exocervix (type I–II)—24; group 2, endocervix (type III)—21)	Ce6 Not Not	661 Not Not Not	Not 4–8	Colposcopy, biopsy, histology, cytology, a PCR analysis of HPV	Group 1: 2.8%—positive; group 2: 3.2%—positive	Group 1: 84%—CR; group 2: 88%—CR
Ivanova [104] 2022 Russia	CIS, MIC T1a1N0M HR-HPV 16, 18, 31,33, 35, 45, 56: 82% (37/45)	Total: 74 (group 1, CIS, exocervix (type I–II)—36; group 2, CIS, endocervix (type III)—34; group 3, MIC—4)	Ce6 Not Not	661 Not Not Not	Not 4–8	Colposcopy, biopsy, histology, cytology, a PCR analysis of HPV	5.1%—positive in 3 months; 0%—positive in 6 and 12 months	Group 1: 96%—CR; group 2: 95%—CR; group 3: 0%
Afanasiev [105] 2022 Russia	CIN II, CIN III, CIS HPV 16, 18, 33, 52	28	Ce6 1.2 mg/kg 3	662 Not 400 Not	Not 1–3	Liquid-based cytology, HPV-testing, colposcopy with acetic test and Schiller’s test	82% (23/28)—negative in 3 months, 93% (26/28)—negative in 36 months	93% (26/28)—CR in 12 months
Gilyadova [106] 2022 Russia	CIN I: 8, CIN II: 10, CIN III: 18, CIS: 8, Microinvasive SCC: 4, SCC: 4 HPV 16: 20, HPV 18: 16, HPV 6: 6, HPV 11: 4, HPV 35: 2, HPV 56: 4	52	Ce6 0.8–1.2 mg/kg 3	660 Cervix: 290, cervical canal: 250 100–250 Not	Not 1–2	Video- and spectral-fluorescence diagnostics, biopsy, histology, colposcopy, cytology, a PCR analysis of HPV	92.3% (48/52)—negative after 1 course; 100% (52/52)—negative after 2 courses	80.8% (42/52)—CR after 1 course; 100% (52/52) CR after 2 courses
Gilyadova [107] 2022 Russia	CIN III: 17, CIS: 28 HPV 16: 18, HPV 18: 14, HPV 33: 6, HPV 11: 2, HPV 35: 3, HPV 56: 2	45	Ce6 1 mg/kg 2	660 Cervix: 290, cervical canal: 250 Cervix: 250–300, cervical canal: 200 Not	Once every 6 weeks 1–2	Video- and spectral-fluorescence diagnostics, biopsy, histology, colposcopy, cytology, a PCR analysis of HPV	100% (45/45)—negative	88.9% (40/45)—CR; 11.1% (5/45)—PR
Gilyadova [108] 2024 Russia	CIN III, CIS HPV 16: (PDT group: 40% (18/45); conization group: 49% (24/49)); HPV 18: (PDT group: 31.1% (14/45); conization group: 20.5% (10/49))	Total: 94 (PDT group: 45, conization group: 49)	Ce6 1 mg/kg 2	660 Cervix: 300, cervical canal: 200–250 Cervix: 350, cervical canal: 250 Not	Once every 6 weeks 1–2	Video- and spectral-fluorescence diagnostics, biopsy, histology, colposcopy, cytology, a PCR analysis of HPV	PDT group: 91.1% —negative after 1 course; conization group: 69.4%—negative after 1 course	PDT group: 86.7% (39/45)—CR, 13.3% (6/45)—PR; conization group: 67.3% (33/49)—CR, 32.7% (16/49)—PR

Abbreviations: HPV—Human Papillomavirus; CIN—Cervical Intraepithelial Neoplasia; HR—High-Risk; CIS—Carcinoma In Situ; MIC—Microinvasive Cancer; SCC—Squamous Cervical Cancer; PDT—Photodynamic Therapy; PS—Photosensitizer; Ce6—Chlorin e6; PCR—Polymerase Chain Reaction; CR—Complete Response; PR—Partial Response. Ce6 (Fotolon and Fotoran) was used as the PS, dissolved in physiological saline and administered to patients at concentrations ranging from 0.8 to 2.5 mg/kg by intravenous infusion over 30 min. Ce6 accumulated in the tissues of the cervix and the cervical canal of the patients for 2 to 4 h.

**Table 4:** Description of articles included in the review (continuation).

Reference Year Country	Localization HPV Type	Numbers of Patients	PS Concentration Accumulation Time, h	Wavelength, nm Power Density, mW/cm^2^ Energy Density, J/cm^2^ Exposure, Min	Repetition Rate Number of Courses	Diagnostics	HPV Outcomes	Lesion Outcomes
Godoy [113] 2013 USA	Cervical, vaginal and anal lesions and other Not	Total: 32 (cervical, vaginal, and anal lesions: 21; other: 11)	Photofrin II 2 mg/kg 48	630 Not Not Not	Not 1–4	Biopsy, histology	Not	Cervical, vaginal, and anal lesions: 24% (5/21)—CR
Choi [114] 2013 Korea	CIN II: 4, CIN III: 22, CIS: 31, AIS: 2 HPV 16: 33, HPV 18: 3, HPV 31: 5, HPV 33: 1, HPV 58: 1, HPV 66: 1	Total: 59 (group 1, PDT: 13; group 2, PDT + LEEP/cone: 15; group 3, PDT within 3 months after LEEP/cone due to positive margin: 25; group 4, PDT due to recurrent CIN at least 12 months after LEEP/cone: 6)	Photogem 2 mg/kg 48	630 150 150 3	Single treatment protocol 1	Papanicolaou smear, colposcopy, HPV test, punch biopsy	89.8% (44/49)—negative after 3 months, 87% (40/46)—negative after 12 months	98.1% (52/53)—CR

Abbreviations: HPV—Human Papillomavirus; CIN—Cervical Intraepithelial Neoplasia; CIS—Carcinoma In Situ; PDT—Photodynamic Therapy; LEEP—Loop Electrosurgical Excision Procedure; PS—Photosensitizer; CR—Complete Response.

**Table 5:** Description of articles included in the review (continuation).

Reference Year Country	Localization HPV Type	Numbers of Patients	PS Concentration Accumulation Time, h	Wavelength, nm Power Density, mW/cm^2^ Energy Density, J/cm^2^ Exposure, Min	Repetition Rate Number of Courses	Diagnostics	HPV Outcomes	Lesion Outcomes
Olejek [118] 2010 Poland	VLS Not	100	5-ALA 5% 3	Halogen light Not Not Not	Once every 2 weeks 10	Biopsy, histology, immunohistochemical staining	Not	61% (61/100)—PR
Osiecka [119] 2012 Poland	VLS Not	1	5-ALA Not 4	630 100 150 Not	Once in 4 weeks, 6 months, 6 weeks, 3–4 months 6	Not	Not	100%—PR after 6 courses
Lei [117] 2016 China	VLS Not	Total: 40 (PDT group: 20; clobetasol propionate group: 20)	5-ALA 10% 3	633 100 100 Not	Once every 2 weeks 4	Photograph, horizontal VAS, PpIX fluorescence imaging	Not	PDT group: 70% (14/20)—CR, 20% (4/20)—PR; clobetasol propionate group: 35% (7/20)—CR, 30% (6/20)—PR
Olejek [120] 2017 Poland	VLS Not	Total: 100 (group 1: 40, group 2: 60)	5-ALA 10% 3	Group 1: 630, group 2: VIS + IRA 750, 580–1400 40–80 100 10–30	Once every 2 weeks 10	Peripheral blood sampling	Not	41% (41/100)—PR; 51% (51/100)—no symptoms; 8% (8/100)—persistent or worsened symptoms
Maździarz [121] 2017 Poland	VLS Not	102	5-ALA 5% 3	590–760 204 120 10	Once every week 10	Biopsy, vulvoscopy	Not	87.25% (89/102)—CR or PR
Osiecka [122] 2017 Poland	VLS Not	11	5-ALA 20% 5	540 85 62.5 2	Once every 2 weeks 3	PDD	Not	Itching (VRS score): 81.8% (9/11)—lack, 9.1% (1/11)—weak, 9.1% (1/11) moderate, 0% (0/11)—severe after 2 months; 72.7% (8/11)—lack, 18.2% (2/11)—weak, 9.1% (1/11)—moderate, 0% (0/11)—severe after 4 months; 63.6% (7/11)—lack, 27.3% (3/11)—weak, 9.1% (1/11)—moderate, 0% (0/11)—severe after 6 months
Lan [123] 2018 China	VLS Not	10	5-ALA 10% 3	635 100 100 20	Once every 2 weeks 3	Biopsy, histology	Not	Itching: 90% (9/10)—CR; 10% (1/10)—PR
Maździarz [81] 2019 Poland	SCC: 2, VLS: 2, genital warts: 1, VIN I: 1, CIN III: 2, CIN I: 2 HPV 16: 5, HPV 31: 1, HPV 42: 1	10	5-ALA 5% 3	590–760 204 120 10	Once every week 10	Colposcopy, vulvoscopy, biopsy, histology, HPV DNA testing	17% (1/6)—negative	90% (9/10)—CR; 10% (1/10)—PR
Li [124] 2020 China	VLS Not	10	5-ALA 20% 3	635 80 80 30	Once every week 4–9	Biopsy, histology	Not	92.31%—CR
Cao [125] 2020 China	VLS Not	1	5-ALA 10% 3	635 200 100 Not	Once every week 3	Biopsy, histology, PDD	Not	100%—CR after 3 courses
Zhang [126] 2020 China	VLS Not	30	5-ALA 20% 3	635 60–90 60–0 20	Once every 2 week 3	Clinical diagnosis, biopsy, histology, HPV detection	Not	90% (27/30)—CR; 10% (3/30)—PR
Zhang [127] 2021 China	VLS Not	30	5-ALA 20% 3	635 60–90 100–150 20	Once every 2 week 3	Biopsy, histology	Not	90% (27/30)—CR; 10% (3/30)—PR
Liu [128] 2021 China	VLS Not	24	5-ALA 20% 3	633 60 Not 30	Once every 2 weeks 6	Dermoscopy	100	100% (24/24)—improvement
Zielińska [129] 2021 Poland	VLS HPV	73	5-ALA 5% 120	630 204 120 10	Once every week 3	Vulvoscopic examination, PDD, biopsy, histology, HPV DNA testing	23% (17/73)—positive, 77% (56/73)—negative	75% (55/73)—CR
Bizoń [85] 2022 Poland	VLS, VIN, CIN, EIN Not	Total: 182 (group 1 (patients with VIN, CIN, EIN: 44; group 2 (patients with family cancer history): 51; group 3 (control): 87)	5-ALA 2 mg/mL 2	630 204 Not 10	Once every week 10	Vulvoscopy, questionnaire	Not	Vulvar changes: (group 1: 21.9%; group 2: 21.2%, group 3: 21.8%); itching: (group 1: 39.3%; group 2: 35.5%, group 3: 42.5%)
Wang [130] 2023 China	VLS Not	31	5-ALA 20% 3	633 60 Not 30	Once every 2 weeks 2	Clinical evaluation, HFUS, biopsy, histopathology	Not	Itching and burning pain: 30% (9/31)—CR after 2 courses
Qu [131] 2024 China	VLS Not	42	5-ALA 20% 3–4	635 60–80 Not 30–40	Once every 10 days 3–6	Assessment of typical clinical symptoms and signs	Not	83.33% (35/42)—CR
Zheng [132] 2024 China	VLS Not	36	5-ALA 20% 3	635 83 100 30	Once every week 6	Biopsy, histology	Not	Itching: 66.76% (24/36)—CR, 27.78% (10/36)—PR; pain: 44.4% (16/36)—CR, 25% (9/36)—PR
Zhang [133] 2024 China	VLS Not	7	5-ALA 20% Not	633 60 108 30	Once every 2 weeks 3	Clinical evaluation, biopsy, histology	Not	Itching: 29% (2/7)—CR; pain: 50% (3/6)—CR
Cao [134] 2024 China	VLS Not	Total: 65 (group 1, early-stage: 44; group 2, late-stage: 21)	5-ALA 20 3	635 60–80 Not 30	Once every 1–2 weeks 3–6	DLQI, FSFI evaluation	Not	Group 1: 90.91% (40/44); group 2: 76.19% (16/21)

Abbreviations: HPV—Human Papillomavirus; VLS—Vulvar Lichen Sclerosus; SCC—Squamous Cervical Cancer; VIN—Vulvar Intraepithelial Neoplasia; CIN—Cervical Intraepithelial Neoplasia; EIN—Endometrial Intraepithelial Neoplasia; PDT Photodynamic Therapy; PS—Photosensitizer; 5-ALA—5-Aminolevulinic Acid; VAS—Visual Analogue Scale; VIS—Visible Light; IRA—Infrared A Light; PpIX—Protoporphyrin IX; PDD—Photodynamic Diagnostics; DNA—Deoxyribonucleic Acid; HFUS—High-Frequency Ultrasound; DLQI—Dermatology Life Quality Index; FSFI—Female Sexual Function Index; PR—Partial Response; CR—Complete Response; VRS—Verbal Rating Scale. Among these 20 analyzed studies, only one reported the detection of HPV 16 in a single patient, while the remaining studies did not identify the presence of HPV in patients with VLS [81]. For PDT, solutions containing 5-ALA at concentrations of 5%, 10%, or 20%, as well as gels or water–oil emulsions, were applied locally to the affected areas using a cotton swab approximately 2–5 h prior to each irradiation session, and subsequently covered with polyethylene film.

**Table 6:** Description of articles included in the review (continuation).

Reference Year Country	Localization HPV Type	Numbers of Patients	PS Concentration Accumulation Time, h	Wavelength, nm Power Density, mW/cm^2^ Energy Density, J/cm^2^ Exposure, Min	Repetition Rate Number of Courses	Diagnostics	HPV Outcomes	Lesion Outcomes
Imbernón-Moya [136] 2016 Spain	VLS Not	8	MAL 160 mg/g 3	630 70 37 9 min 45 s	Once every 6–12 months 1–3	VAS, DLQI, Biopsy, Histology	Not	Significant improvements in symptoms of the disease and quality of life in all patients

Abbreviations: HPV—Human Papillomavirus; VLS—Vulvar Lichen Sclerosus; PS—Photosensitizer; MAL—Methylaminolaevulinate. VAS—Visual Analogue Scale; DLQI—Dermatology Life Quality Index.

## Supplementary Materials

The following supporting information can be downloaded at: https://www.mdpi.com/article/10.3390/cancers17152421/s1, Table S1: Description of articles included in the review.

## Abbreviations

The following abbreviations are used in this manuscript:

PDT	Photodynamic Therapy
PS	Photosensitizer
PSs	Photosensitizers
WHO	World Health Organization
HPV	Human Papillomavirus
LCs	Langerhans Cells
IL-10	Interleukin-10
VLS	Vulvar Lichen Sclerosus
DNA	Deoxyribonucleic Acid
mRNA	Messenger Ribonucleic Acid
LEEP	Loop Electrosurgical Excision Procedure
5-ALA	5-Aminolevulinic Acid
EPR	Enhanced Permeability and Retention
Ce6	Chlorin e6
PpIX	Protoporphyrin IX
ROS	Reactive Oxygen Species
TAMs	Tumor-Associated Macrophages
CIN	Cervical Intraepithelial Neoplasia
CIS	Carcinoma In Situ
CCA	Cervical Condylomata Acuminata
LR-	Low-Risk
HR-	High-Risk
VIN	Vulvar Intraepithelial Neoplasia
EIN	Endometrial Intraepithelial Neoplasia
TCT	ThinPrep Cytology Test
PCR	Polymerase Chain Reaction
CR	Complete Response
PR	Partial Response
HAL	Hexaminolevulinate
MAL	Methylaminolaevulinate
CI	Confidence Interval
MIC	Microinvasive Cancer
SCC	Squamous Cervical Cancer
DAMPs	Damage-Associated Molecular Patterns
AIS	Adenocarcinoma In Situ
DCs	Dendritic Cells
VIS	Visible Light
IRA	Infrared-A Light
PDD	Photodynamic Diagnostics
HFUS	High-Frequency Ultrasound
DLQI	Dermatology Life Quality Index
FSFI	Female Sexual Function Index
VRS	Verbal Rating Scale
VAS	Visual Analogue Scale
MVD	Microvessel Density
ANA	Antinuclear Antibodies
HDB	Hypoechoic Dermal Band
MBP	Myelin Basic Protein
NIR	Near-Infrared
ICG	Indocyanine Green
HeLa	Henrietta Lacks
HYP	Hypericin
MB	Methylene Blue
HA	Hyaluronic Acid
18F-FDG	2-Fluoro-2-Deoxy-D-Glucose
VP	Verteporfin
